# A Genome-Wide RNAi Screen Reveals MAP Kinase Phosphatases as Key ERK Pathway Regulators during Embryonic Stem Cell Differentiation

**DOI:** 10.1371/journal.pgen.1003112

**Published:** 2012-12-13

**Authors:** Shen-Hsi Yang, Tuzer Kalkan, Claire Morrisroe, Austin Smith, Andrew D. Sharrocks

**Affiliations:** 1 Faculty of Life Sciences, University of Manchester, Manchester, United Kingdom; 2 Wellcome Trust–Medical Research Council Stem Cell Institute, University of Cambridge, Cambridge, United Kingdom; Friedrich Miescher Institute for Biomedical Research, Switzerland

## Abstract

Embryonic stem cells and induced pluripotent stem cells represent potentially important therapeutic agents in regenerative medicine. Complex interlinked transcriptional and signaling networks control the fate of these cells towards maintenance of pluripotency or differentiation. In this study we have focused on how mouse embryonic stem cells begin to differentiate and lose pluripotency and, in particular, the role that the ERK MAP kinase and GSK3 signaling pathways play in this process. Through a genome-wide siRNA screen we have identified more than 400 genes involved in loss of pluripotency and promoting the onset of differentiation. These genes were functionally associated with the ERK and/or GSK3 pathways, providing an important resource for studying the roles of these pathways in controlling escape from the pluripotent ground state. More detailed analysis identified MAP kinase phosphatases as a focal point of regulation and demonstrated an important role for these enzymes in controlling ERK activation kinetics and subsequently determining early embryonic stem cell fate decisions.

## Author Summary

Embryonic stem cells and induced pluripotent stem cells represent potentially important therapeutic agents in regenerative medicine. Manipulation of these cell types could allow us to replace dead or diseased cells in our bodies and hence potentially provide a solution to a wide range of medical problems. However, before we can perform such manipulations, we need to understand how the stem cells are wired so that we are able to re-wire them in a logical way to produce the desired cell types. Here we have attempted to understand this wiring by using an RNAi screen in which each individual component of the cell is systematically removed and the consequences on cellular fate determined. We have identified hundreds of genes that are required for efficient loss of stem cell characteristics and hence conversion into other cell types. By studying a subset of these genes, we have been able to show that many converge on two related negative regulators of one of the key pathways that act to promote loss of stem cell identity. These negative regulators, Dusps, normally limit the ability of stem cells to change their function and hence be converted to different cell types.

## Introduction

Embryonic stem cells and induced pluripotent stem cells (iPS cells) are currently generating intense interest due to their potential therapeutic roles in regenerative medicine (reviewed in [1]). We are beginning to understand the rules governing the establishment and maintenance of the pluripotent state and, in particular, the signaling and transcriptional networks which define this state (reviewed in [2]–[3]). A number of genome-wide si/shRNA screens have been instrumental in deciphering these networks [4]–[6]. In contrast, less attention has been directed towards understanding how embryonic stem cells lose their pluripotency and begin to differentiate.

Mouse embryonic stem cells can be maintained in a pluripotent state by culturing under a variety of defined conditions (reviewed in [7]). Traditionally, these cells are cultured in medium containing serum and the cytokine leukaemia inhibitory factor (LIF) [8]–[9]. However, more recently, it was demonstrated that mouse embryonic stem cells can be maintained in a pluripotent ground state by using two specific protein kinase inhibitors (known as “2i” conditions) which target the ERK pathway component MEK and glycogen synthase kinase (GSK3) ([10]; reviewed in [11]). Removal of these two inhibitors promotes exit from the naïve ground state. These studies therefore revealed an important role for the ERK and GSK3 pathways to enter into lineage commitment (reviewed in [12]). Moreover, the suppression of ERK signalling in the mouse embryo is sufficient to expand the pluripotent compartment in the early mouse embryo [13] and can enhance the efficiency of iPS cell generation by promoting completion of reprogramming [14]–[15]. Importantly, the same pathways may operate in a functionally analogous manner in human pluripotent stem cells that have been genetically manipulated [16]–[17]. The ERK pathway has previously been shown to trigger mouse ES cell differentiation [18]–[19] and is implicated in numerous developmental processes (reviewed in [20]) in addition to playing an important role in a variety of different stem cell types (reviewed in [21]). Less is known about GSK3 function in development and stem cell biology and the role for GSK3 is usually attributed to its ability to regulate β-catenin stability and hence limit the responses to Wnt pathway signalling (reviewed in [11], [22]). Recently, a β-catenin-dependent mode of action has been demonstrated for GSK3 in the context of mouse embryonic stem cells, although this mode of action is not sufficient to explain all the effects of GSK3 signalling in this context ([23]–[24]; reviewed in [25]).

One major function of ERK MAP kinase signalling, is to orchestrate gene expression programmes in the cell. In particular, this pathway directly targets a number of transcription and chromatin regulators and thereby controls their activities (reviewed in [26]–[27]). However, which of the ERK targets are important in embryonic stem cell differentiation are unknown. It is also unclear how the canonical ERK pathway is controlled in these cells. In this study, we took advantage of the fact that the combinatorial use of ERK pathway and GSK3 inhibitors maintains mouse embryonic stem cell pluripotency [10] and carried out a genome-wide siRNA screen to identify regulators and mediators of these pathways that influence the exit from pluripotency. This has led to the identification of over 400 genes whose functions are required for efficient embryonic stem cell differentiation away from the pluripotent ground state. The vast majority of these genes have not previously been implicated in this process; therefore our study provides an important new resource for the community. Moreover, further downstream analysis has partitioned these genes into classes that functionally interact with the ERK and/or GSK3 pathways and has revealed an important role for MAP kinase phosphatases in controlling embryonic stem cell fate.

## Results

### An RNAi screen for genes required for ERK/GSK3-mediated ES cell differentiation

**Figure 1:**
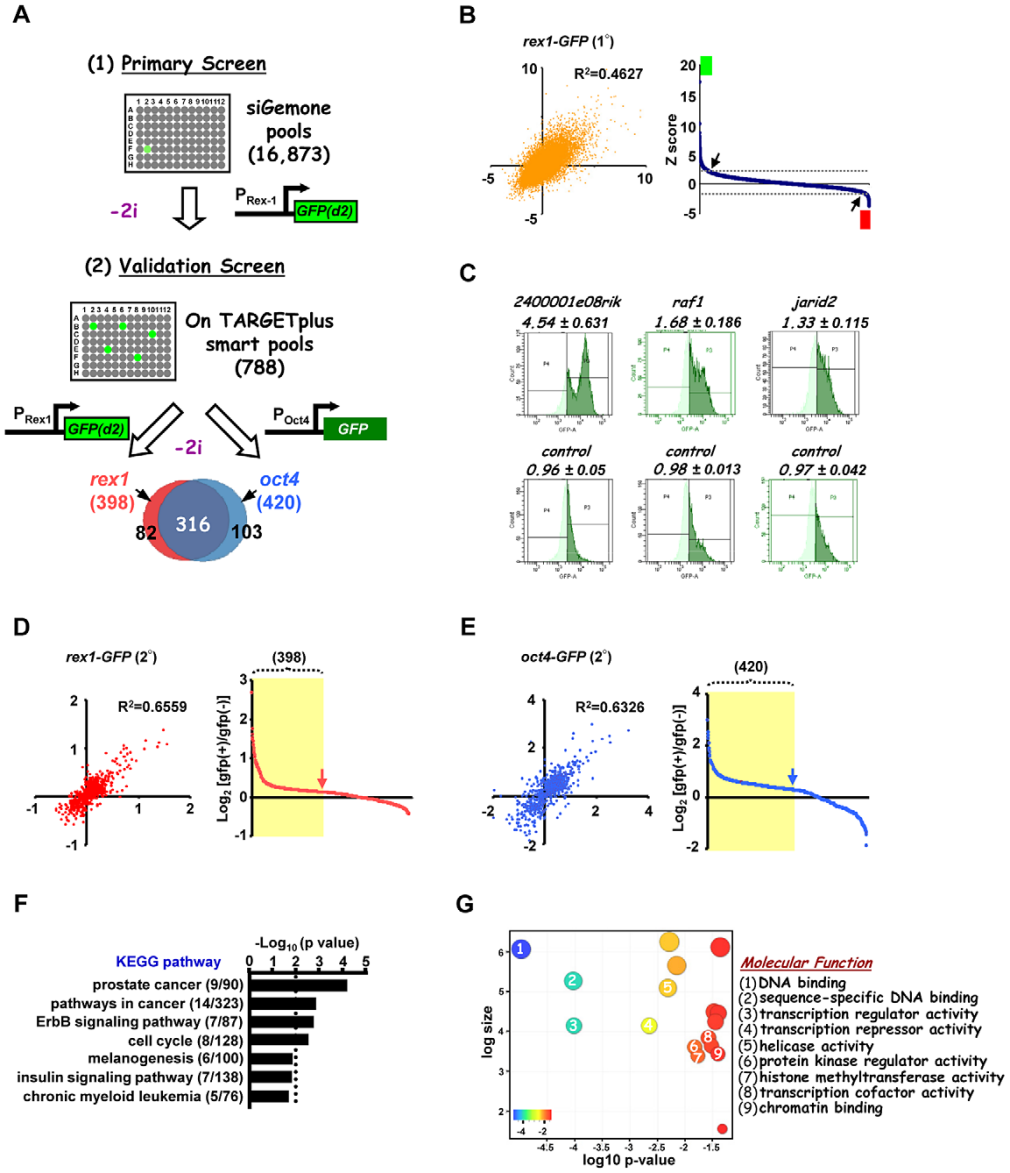
A genome-wide RNAi library screen identifies factors involved in signal-dependent embryonic stem cell differentiation. (A) Schematic representation of primary screen and secondary validation screens using mouse embryonic stem cell lines containing *GFP* reporter constructs under the control of either the endogenous *rex1* (*Rex1*GFPd2) and/or *oct4* (*Oct4*GFP) promoters. “−2i” indicates that the two kinase inhibitors (CHIR99021 and PD0325901) were removed for 28 hrs (*Rex1*GFPd2 cells) and 72 hrs (*Oct4*GFP cells) before quantifying the GFP-positive population of cells. The Venn diagram shows 316 high confident hits resulting from the overlap of both validation screens. (B) The z-score of each of two biological replicates from the primary screens are plotted against each other (left panel). The average of ranked z-scores from the knockdown of individual siRNA pools is shown (right panel). The arrows indicate the z-score threshold (2 and −2). The green and red boxes mark the hits with z-score >2 and <−2, respectively. (C) Representative FACS profiles from control non-targeting and positive siRNA hits from the primary *rex1-GFP(d2)* screen. The high GFP expressing population is depicted in dark green and numbers above each graph are the corresponding GFP high/GFP low ratios. (D and E) The log_2_ ratio of GFP(+)/GFP(−) of each of the two biological replicates are plotted against each other in either the *rex1-GFP(d2)* (D) or *oct4-GFP* (E) validation screens (left panels) and graphical representations of the ranked log_2_ values of these ratios from the average of two independent experiments upon knockdown of individual genes are shown (right panels). The yellow shaded boxes indicate the positive hits which scored as a GFP(+)/GFP(−) ratio above 1.25× standard deviation of the controls in each of the sub-screens. (F and G) Enriched KEGG (F) and “molecular function” level GO terms (G) amongst the high confidence hits identified in both of the secondary validation screens.

To identify the programme of genes involved in the loss of pluripotency and subsequent differentiation of embryonic stem cells, a genome-wide RNAi screen was performed using E14Tg2a mouse ES cells which are engineered to express an unstable version of GFP from the endogenous *rex1* (also known as *zfp42*) locus. This reporter gene is regulated in an analogous manner to endogenous *rex1* [23] and provides a convenient readout for the loss of a naieve pluripotent stem cell marker Rex1 [28] (reviewed in [11]). *Rex1*GFPd2 ES cells were maintained in media containing MEK and GSK inhibitors (2i) to maintain their ES cell status and treated with siRNAs pools targeting ∼17,000 individual genes. After 24 hrs, cells were exchanged into fresh media lacking these inhibitors and the levels of GFP in each cell were assessed over time (Figure 1A). A gradual loss of GFP expression occurred upon inhibitor withdrawal over a ∼2 day time period, with conversion of the majority of cells to low expression (1A). We wanted to conduct the screen at the earliest possible time point to maximise the chances of detecting genes directly involved in the exit from pluripotency rather than secondary effectors. The control siRNAs for *fgf4* and *gsk3β* both significantly reduced GFP loss at 27–30 hrs (Figure S1B). Therefore we monitored the ratio of cells expressing high and low levels of GFP at this time point. siRNAs were scored as positive hits when this ratio increased by more than two standard deviations (SD) above the mean of all siRNAs on each plate. A conservative threshold was selected at this stage to be more inclusive before further downstream validation was performed. This led to the identification of 792 siRNAs that delayed the loss of GFP expression, and hence target genes potentially involved in promoting pluripotency loss and/or cell differentiation (Figure 1B; Table S1A). Examples, include *2400001e08rik*, *raf1* and *jarid2* (Figure 1C; Figure S2A–S2D, left panels). Importantly, this primary screen identified RNAi pools targeting *nras*, *raf1* and *gsk3β*, as would be expected due to their known roles in the ERK and GSK3 pathways. Moreover, further validation of the efficacy of our screen was demonstrated by the identification of a large number of siRNAs targeting genes encoding proteosomal proteins, as would be expected due to the subsequent increased half-life of the unstable GFP protein used as a readout in these assays. In addition, this primary screen also revealed 130 siRNAs that accelerate the loss of GFP expression and hence target genes that function to maintain pluripotency and/or inhibit cell differentiation including known effectors such as *esrrb, stat3, and ctr9* [4], [29]–[30](Figure 1B; Figure S2E; Table S2). Furthermore, several of genes identified in our screen in this category were also identified in other screens designed to identify genes required for pluripotency [4]–[5], [31]–[33], including *stat3* and *smc1a* (both identified in 2 and 3 additional screens, respectively) (Table S3). As our primary interest was on the mechanisms of escape from the pluripotent ground state rather than the maintenance of pluripotency, we subsequently focussed on genes that were required for modulating the onset of differentiation. Two secondary screens were performed with a different set of siRNA pools targeting the genes identified in the primary screen and either the same reporter cells (ie *Rex1*GFPd2) or ES cells containing an alternative reporter gene, where *GFP* is instead driven by the *oct4* (also known as *pou5fl*) promoter, thereby providing an independent readout for the loss of pluripotency (Figure 1A; Figure S1C). These screens gave rise to 398 and 420 positive hits respectively, and 316 of these siRNAs scored positive in both secondary screens (Figure 1A, 1D and 1E; Figure S2A–S2D, Table S1B and S1C). These 316 siRNAs therefore define a high confidence dataset of genes that are required for the efficient loss of pluripotency and/or promoting the onset of differentiation of ES cells. A number of these genes have already been implicated in embryonic stem cell differentiation control including *tcf7l1(tcf3)*, *jarid2*, and *dpy30* [34]–[37] (Table S4) further supporting the quality of our dataset. Moreover, comparisons to other RNAi and overexpression screens performed on mouse ES cells [4], [31]–[33], [38] identified several genes in common, including *jun* and *mbd3* which were both identified in two of these screens in addition to our own (Table S3). However, the vast majority of genes we have identified here, have not been previously implicated in controlling the escape from the pluripotent ground state. To assess the types of biological processes and potential mechanisms of actions of these 316 genes, gene ontology (GO) analysis was performed and prominent terms identified included a number of signalling pathways and also genes encoding transcriptional regulators (Figure 1F and 1G; Figure S3). Thus cellular signalling events and subsequent gene expression control appear to play prominent roles in the early events associated with ES cell differentiation.

### Distinct groups of genes are required for the action of the ERK and GSK3 signalling pathways

**Figure 2:**
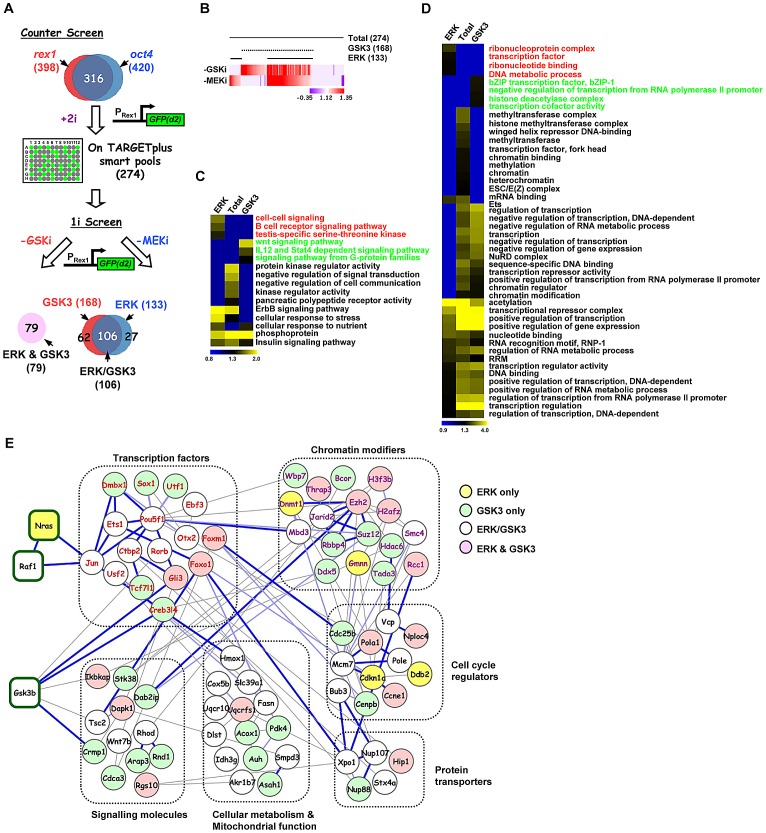
Secondary screening and association of genes to the ERK and/or GSK3 pathways. (A) Schematic representation of the strategy used to stratify the high confidence positive hits via either the ERK and/or GSK3 pathways (1i-screens) using the *rex1-GFP* reporter system. A counter screen was performed in the presence of the two kinase inhibitors (CHIR99021 and PD0325901)(“+2i”) and remaining hits were tested when either the GSK3 inhibitor (CHIR99021; -GSKi) or the MEK inhibitor (PD0325901; -MEKi) was withdrawn. The Venn diagram illustrates the four different hit categories. (B) Heatmap summary depicts the stratification of the hits according to their effects on the GFP(+)/GFP(−) ratio upon withdrawal of the GSK3 inhibitor (-GSK3i) or MEK inhibitor (-MEKi). The numbers of genes in each category are indicated. The colour scale represents the ratio of high to low GFP expressing cells for each siRNA pool in each of the “1i” screens. (C and D) Heatmaps of the enriched GO terms identified for genes corresponding to hits specific to the total dataset from the “2i” screen (274), or hits from the “1i” screens; ERK (133), or GSK3 (168) pathways. Each GO term is scored by −log_10_(P-value). The associated GO term descriptions are indicated (the GO terms enriched in only the ERK or GSK3 categories are indicated in red or green font respectively). Distinct functional groups corresponding to terms associated with cell signaling (C) and gene expression (D) are manually clustered. (E) STRING network analysis of the core network formed by the 274 genes associated with signal-dependent loss of pluripotency and promoting early differentiation processes in the mouse embryonic stem cells. Genes are grouped according to common biological processes. The coloured lines of edges represent confidence scores of interconnectivity. Dark blue lines represent 0.8–1, light blue lines represent 0.6–0.8, and light grey lines represent 0.4–0.6 confidence levels, respectively.

Having established the core network of genes working in concert with the GSK3 and ERK pathways we wanted to discover the relative contributions of these genes to the actions of the individual pathways. First we performed a counter screen in the presence of both pathway inhibitors (“+2i”) to eliminate siRNAs which promoted accumulation of GFP in the cells irrespective of the activity of the ERK and GSK3 pathways (Figure 2A). This eliminated a further 42 siRNAs, including 14 that targeted proteosomal components and hence stabilised the GFP (Table S5). This left 274 siRNAs which define genes required for efficient signal-dependent loss of pluripotency and the onset of differentiation. The differentiation of ES cells away from pluripotency is maximally promoted by removing inhibitors of both GSK3 and the ERK pathway. However, the removal of a single inhibitor permits ES cell differentiation and loss of Rex1-GFP signal, albeit with delayed kinetics (Figure S4). We took advantage of this to partition our dataset and identify genes whose functions are specifically required for differentiation driven by either the ERK pathway or the GSK3 pathway alone. siRNAs targeting the genes constituting the high confidence data set from the “2i” withdrawal screens were tested for their effect on Rex1-GFP loss upon single inhibitor withdrawal (ie “1i” withdrawal screens; Figure 2A). Of the 274 siRNAs tested, 133 delayed GFP loss upon withdrawal of the MEK inhibitor and 168 upon withdrawal of the GSK3 inhibitor. Amongst these, 106 were in common. A further 79 siRNAs had no effect on Rex1-GFP expression under either condition (Figure 2A and 2B; Figure S5). Thus there are four functionally distinct classes of hits identified that are involved in promoting the onset of differentiation: (i) in the context of the ERK pathway (“ERK only hits” eg *nras*, Figure S2A; identified upon MEK inhibitor withdrawal only); (ii) in the context of the GSK3 pathway (“GSK only hits” eg *dmbx1*, Figure S2B; identified upon GSK3 inhibitor withdrawal only); (iii) in the context of either pathway (“ERK/GSK hits” eg *jun*, Figure S2C; identified upon GSK3 or MEK inhibitor withdrawal); and (iv) in the context of both pathways together (“ERK and GSK hits” eg *gli3*, Figure S2D; no effect when either inhibitor is withdrawn). Next to gain an insight into how the ERK and GSK3 pathways might function in the context of embryonic stem cells, we used gene ontology analysis to determine whether different groups of genes identified from the single inhibitor (“1i”) screens are associated with different biological processes. Generally, the enriched GO terms for the genes from the initial 2i screen closely resemble those enriched in the “GSK” dataset (Figure S6). However, closer inspection of the data revealed enriched GO terms that are more specific for genes which were associated with either the ERK or the GSK3 pathway, thereby revealing functionally distinct contributions of these pathways to the exit from pluripotency (Figure 2C and 2D; Figure S7A–S7D). For example, genes associated with either the ERK or GSK3 pathways are enriched in different signalling pathways (Figure 2C) and a number of terms associated with mitochondrial function are preferentially enriched in the genes associated with the GSK pathway (Figure S7C). However, other groups of GO terms were identified with generally high enrichment for genes associated with both the GSK and the ERK pathways. This is typified by a large number of GO terms associated with transcriptional control (Figure 2D). Weaker enrichment of specific terms could be discerned for genes functionally associated with either the ERK or the GSK pathways (Figure 2D).

We then created a network out of the genes from the high confidence dataset identified in the “2i” screen based on previous knowledge of physical and functional interactions. Functionally related subnetworks could be identified, two of the most prominent of which are composed of genes encoding proteins associated with regulating chromatin modifications and sequence-specific DNA binding transcription factors (Figure 2E; Figure S8A). These genes showed strong interconnectivities with the rest of the network as might be expected from their regulatory functions. Although only a limited number of connections between ERK and GSK3 signalling pathway components identified in the screen were revealed during network construction, these connections are made to transcription and chromatin regulators associated with the correct respective pathways (eg Jun is connected to the Ras pathway and Gli3 is connected to Gsk3β; Figure 2E; Figure S8B).

In summary, by comparing single inhibitor assays, we have been able to subcategorise the genes required for embryonic stem cell differentiation and tentatively assign them to mediating or regulating the effects of either the ERK pathway, or the GSK3 pathway or both. Each pathway appears to require genes associated with overlapping and yet distinct biological processes.

### Functional dissection of genes associated with ERK pathway signalling

**Figure 3:**
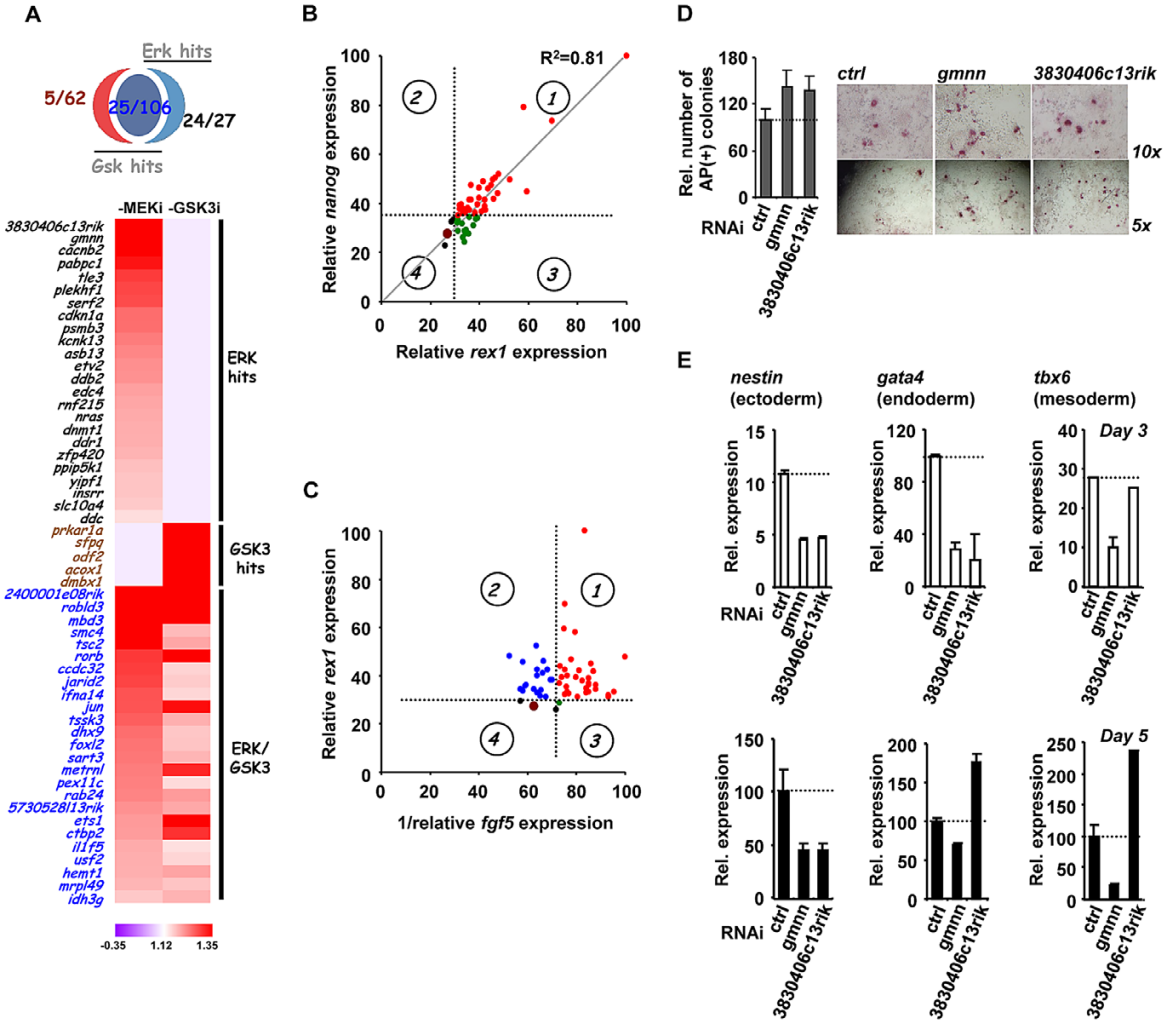
The role of ERK pathway-specific hits in the expression of pluripotency and early differentiation marker genes. (A) Venn diagram (top) and heatmap summary (bottom; see Figure 2B for details) illustrating the number and a list of selected screen hits used in the subsequent studies. These selected hits are distributed within three categories as indicated on the heatmap. The colour scale represents the ratio of high to low GFP expressing cells for each siRNA pool in each of the “1i” screens. (B) *rex1* (x-axis) and *nanog* (y-axis) mRNA expression levels following 2i withdrawal for 36 hrs are plotted upon knockdown of individual genes (see Figure S9B for details). Data are shown for each siRNA duplex relative to the maximal expression exhibited in the presence of an siRNA pool (taken as 100). Dotted lines represent the expression values >2 standard deviations above the mean of the negative control siRNAs. Red dots represent siRNA duplexes which promote elevated expression of both genes (quadrant 1), whereas green (quadrant 3) and black (quadrant 4) dots represent siRNAs that cause changes at or below this threshold cut-off value for only one gene. The brown dot represents the negative control siRNAs. (C) *rex1* (y-axis) and the reciprocal of *fgf5* (x-axis) mRNA expression levels upon 2i withdrawal for 36 hrs and 48 hrs, respectively, are plotted following knockdown of individual genes (see Figure S9B and C for details). The labeling is as indicated in (B), except that red dots represent siRNA duplexes which promote elevated expression of *rex1* and lower levels of *fgf5* (quadrant 1). Blue dots (quadrant 2) represent siRNAs that cause elevated *rex1* expression but fail to show reductions in *fgf5* expression. (D) Alkaline phosphatase staining of *Rex1*GFPd2 ES cells following treatment of cells with siRNAs against *gmnn* or *3830406c13rik* or a non-targeting control (ctrl) and release from “2i” for 5 days. Data are means ± SEM (n = 2) (E) RT-PCR analysis of the expression of the indicated lineage marker genes following treatment of cells with siRNAs against *gmnn* or *3830406c13rik* or a non-targeting control (ctrl) and release from “2i” for 3 (top) or 5 days (bottom). Data are presented as means ± SEM (n = 2).

Our RNAi screen identified genes belonging to many functionally related categories and they are potentially involved in many biological processes. However, to begin to understand the roles of the genes we have identified in controlling the loss of pluripotency and subsequent differentiation, we decided to focus mainly on the genes which were required for ERK-mediated differentiation as this pathway has a well established role in triggering mouse ES cell differentiation [18]–[19]. The majority of “ERK only” genes and a subset of “ERK/GSK” genes were taken for further investigation alongside several control genes from the “GSK only” hits (Figure 3A). The relative strength of the effect of the knockdown of each gene in the context of the “1i” screens is illustrated in Figure 3A. First we validated the roles of these genes by using RT-qPCR to monitor the loss of the pluripotency markers *rex1* and *nanog* and the appearance of the early differentiation marker *fgf5*. The majority of the siRNAs tested showed increased *rex1* and *nanog* expression relative to control siRNAs upon “2i” withdrawal (Figure 3B; Figure S9B). Importantly, an excellent correlation was observed between effects on *rex1* and *nanog* expression (Figure 3B; R^2^ = 0.81). Conversely, more than half of the siRNAs tested reduced the accumulation of *fgf5* mRNA (Figure S9C). However, there was generally reduced concordance between the severity of the effects on *fgf5* and *rex1* (Figure 3C) or *fgf5* and *nanog* (Figure S9D) expression. For example depletion of *jarid2* and *pabpc1* causes some of the largest effects in maintaining *rex1* expression but has no effect on reducing *fgf5* accumulation. Conversely, reductions in *ets1* and *dmbx1* limit *fgf5* expression while having only a small effect on *rex1* expression. Nevertheless, a group of siRNAs can be identified that limit the loss of *rex1* expression and show reduced accumulation of *fgf5* (Figure 3C; quadrant 1) and hence have effects on both loss of naive pluripotency and the onset of differentiation. In contrast, there is another large group of genes that appear to affect pluripotency status but have little effect on the onset of early differentiation (Figure 3C, quadrant 2). It is unclear why this occurs but it might reflect that although individual siRNAs promote retention of pluripotency, they might also trigger the activation of subsets differentiation markers, thus the two processes need not be tightly linked. To extend the analysis of differentiation events, we focused on the two of the top hits attributed to ERK signalling, *gmnn* and *3830406c13rik*, and also asked whether the appearance of markers of the three embryonic cell lineages was affected. First we determined whether pluripotent cells remained in the population by alkaline phosphatase staining. Increased numbers of alkaline phosphatase stained cells were identified 5 days after “2i” withdrawal upon depletion of either gene, confirming their importance for escape from the pluripotent ground state (Figure 3D). Depletion of *gmnn* caused reductions in the expression of all three lineage markers at both 3 and 5 days following “2i” withdrawal, consistent with a general role in regulating the escape from the pluripotent ground state (Figure 3E). Similarly, depletion of *3830406c13rik*, caused reduced expression of all three markers at day 3 (albeit only marginally for *tbx6*), and reduced levels of *nestin* after 5 days (Figure 3E). However, increased expression of *gata4* and *tbx6* was observed at this later timepoint, suggesting a lineage specific role for this gene. Thus, the contributions of individual genes identified in our screen towards individual lineage commitment are likely complex. In summary, the use of marker genes allows us to further validate the hits in our screen, although the effects of depleting individual genes on the loss of naive pluripotency and/or differentiation vary according to the gene involved.

**Figure 4:**
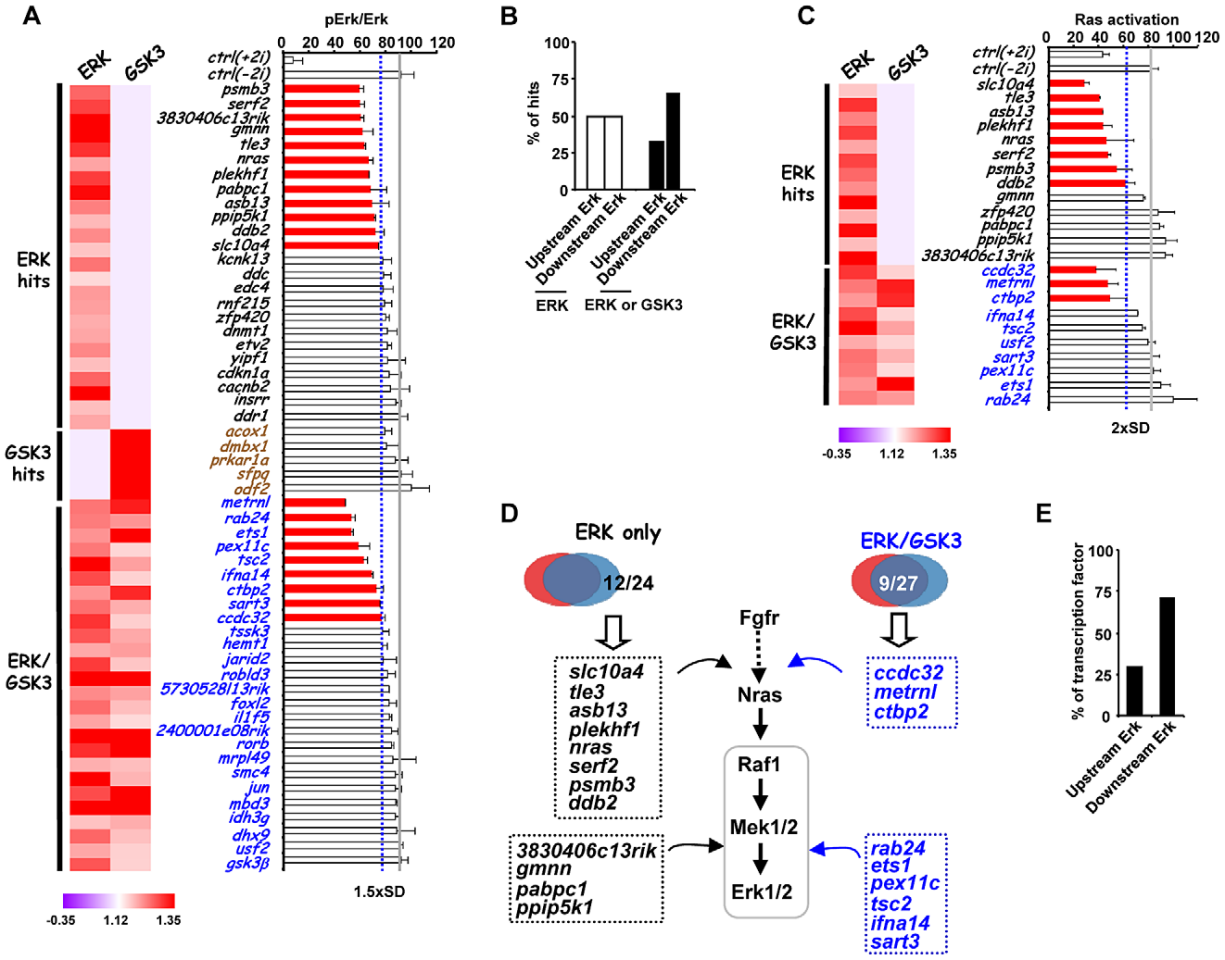
Association of siRNA screen hits with the ERK signaling pathway. (A) ERK activation levels following 2i withdrawal (−2i) for 20 mins are plotted as the ratio of phospho-ERK2 and ERK2 signals upon depletion of selected genes as indicated. The blue dashed line indicates the threshold level (1.5× SD above the mean of the negative controls) and levels below this are indicated by red bars. The average activity in the presence of control siRNA (ctrl) is shown by the solid grey line and data are plotted relative to the siRNA giving the highest levels of phospho-Erk (taken as 100). The data are presented as means ± SEM and are the average of three biological replicates (n = 3). A heatmap summary of the effect of each siRNA duplex in the “1i” screens is shown on the left. (B) Summary of the points of action of the siRNA screen hits with respect to the ERK pathway. Genes are partitioned according to which class of siRNA hits they belong. (C) Ras activity levels upon depletion of the indicated genes upon 2i withdrawal (−2i) for 2 mins. The blue dashed line indicates the threshold level (2× SD above the mean of the negative controls) and levels below this are indicated by red bars. The average activity in the presence of control (ctrl) siRNA is shown by the solid grey line and data are plotted relative to the siRNA giving the highest levels of Ras activity (taken as 100). Data are presented as means ± SEM and are the average of three biological replicates (n = 3). A heatmap summary of the effect of each siRNA duplex in the “1i” screens is shown on the left. (D) Summary diagram illustrating the point of action of upstream ERK effectors in the ERK pathway as either upstream of Ras or between Ras and Erk. The hit lists are grouped into the ERK-unique or ERK/GSK-shared hit categories. (E) Summary of the points of action of genes encoding transcriptional regulators respect to the ERK pathway.

Next, to further investigate the function of the hits identified in our screen, we investigated how this subset of genes impacted on ERK pathway regulation and function. In theory, genes might act to control ERK pathway activity or alternatively might mediate the effects of ERK pathway signaling. Therefore as a first step to partition genes as acting up or downstream of ERK, we used western blotting to monitor the active phosphorylated form of ERK (Figure 4A, Figure S10). Using this assay, siRNAs targeting 21 different genes were identified as upstream regulators of ERK. Importantly, none of the “GSK3 only” hits affected ERK activation, further validating our partitioning of the data (Figure 4A; Figure S10). Furthermore, while “ERK only” hits are partitioned evenly as acting up and downstream of ERK activation, the “ERK/GSK” hits are more prominent downstream of ERK (Figure 4B), as might be expected for genes which are important for GSK-mediated differentiation when ERK signaling is inhibited. To further delineate their point of action, we then tested the subset of siRNAs which acted upstream of ERK for their effects on Ras activation by an ELISA-based assay (Figure 4C). Eleven genes were identified whose point of action is upstream of both Ras and ERK (Figure 4C; Figure S11). Importantly, one of these genes was *nras* itself. These assays therefore enabled us to position genes from the “ERK only”, and “ERK/GSK” datasets at different points in the ERK pathway, either acting upstream of Ras eg *plekh1* or on the core pathway downstream from Ras (Figure 4D). The rest of the genes analysed appear to act downstream from ERK and hence are likely mediators of ERK pathway function. Interestingly, transcription factors are over-represented in the subgroup of genes which act downstream of ERK (Figure 4E), in keeping with the known major role of ERK signalling in controlling gene expression programmes (reviewed in [26]–[27]). Together, these findings indicate that we have identified groups of genes which affect either signalling through the ERK pathway and/or the downstream consequences of ERK activation.

While in this study we have focussed on studying genes which affect escape from pluripotency, and are associated with the ERK and GSK3 pathways, it is likely that many of the genes we have identified might also play a more general role in controlling stem cell pluripotency. Indeed, several genes identified in our study were also identified previously in other siRNA screens conducted in cells maintained in the presence of serum and LIF rather than the “2i” conditions we used (Table S3). To investigate this further, we tested 8 genes for their role in escape from pluripotency in *Rex1*GFPd2 ES cells maintained in serum and LIF and induced to differentiate by withdrawal of LIF. Depletion of three of these genes, *otx2*, *etv5* and *mbd3*, caused an increased retention of *rex1* promoter-driven GFP expression, consistent with a disruption in escape from pluripotency (Figure S12). Thus, it is likely that many of the genes we have identified in this screen will play a more general role in controlling cell fate decisions in ES cells maintained in serum plus LIF or “2i” conditions.

### Dual specificity phosphatases are key regulators of ES cell differentiation

A group of 10 genes was identified which acted downstream of Ras but affected ERK phosphorylation levels and hence ERK activity (Figure 4D). To further probe the point of action of these genes, we tested MEK activation levels following their depletion but saw little difference (data not shown). Next, we therefore focussed on MAP kinase phosphatases (also known as dual specificity phosphatases [DUSPs]), and hypothesised that increases in the levels and/or activity of these enzymes might be responsible for the reduced ERK activation that we observed and consequent effects on embryonic stem cell differentiation.

**Figure 5:**
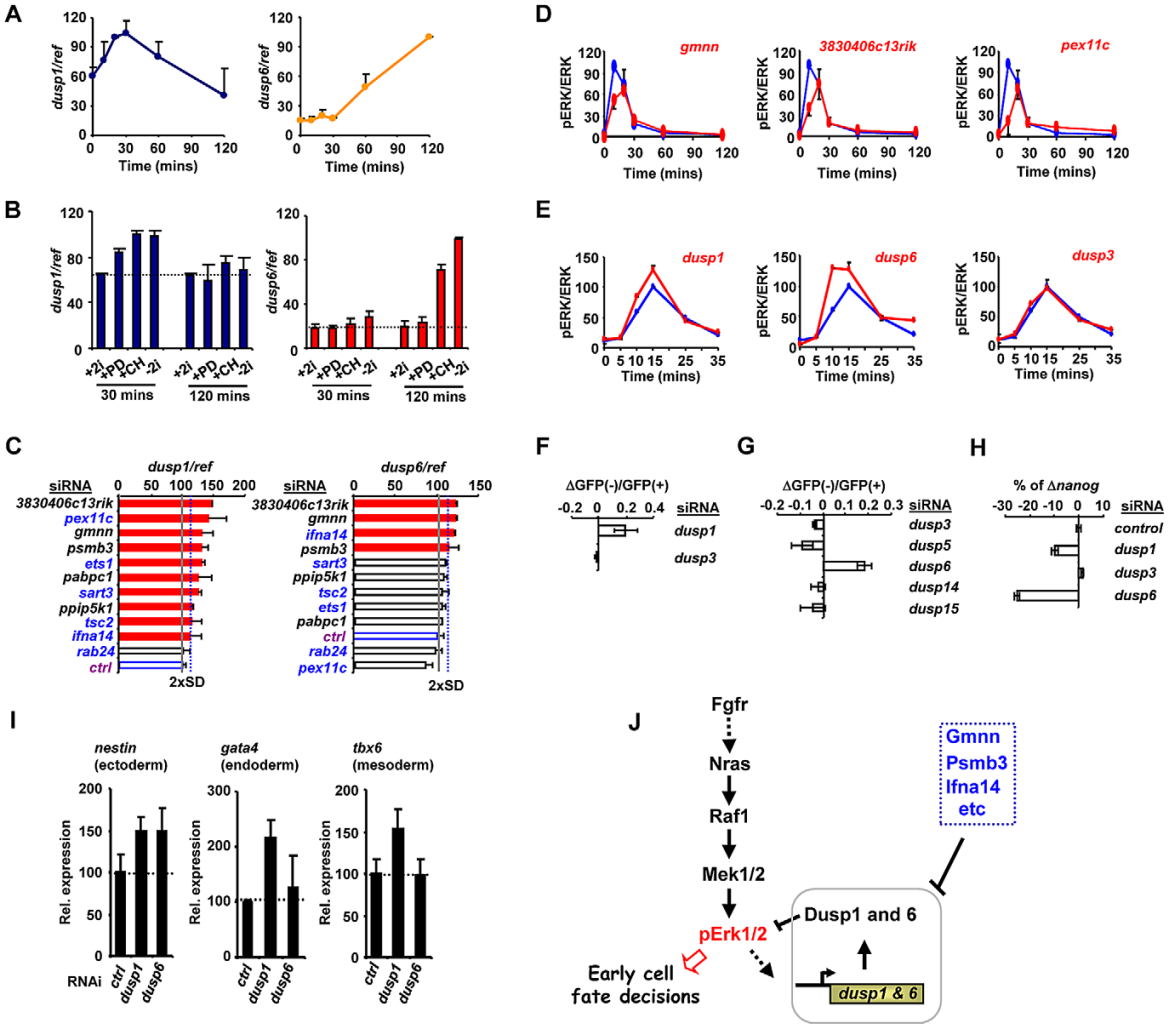
The regulation of Dusp1 and Dusp6 activity and ES cell differentiation. (A–C) RT-qPCR analysis of *dusp1* and *dusp6* mRNA expression in mouse ES cells. Data are normalised by the average values of three reference genes (ref) and are presented as means ± SEM and are the average of three biological replicates (n = 3). (A) The kinetics of *dusp1* and *dusp6* expression at the indicated times following 2i withdrawal. (B) The effects of the indicated inhibitors, either alone or in combination, on the expression of *dusp1* and *dusp6* at the indicated times following inhibitor withdrawal. (C) The effects of depletion of the indicated genes on *dusp1* and *dusp6* mRNA expression in the presence of 2i. The blue dashed line indicates the threshold level (2× SD above the mean of the negative controls) and levels below this are indicated by red bars. The average activity in the presence of control siRNA (ctrl) is shown by the solid grey line (taken as 100). (D and E) Active ERK levels were determined by the ratio of phospho-ERK (pERK)/total ERK (ERK) levels at the indicated times following 2i release in the presence of the indicated siRNAs (red lines) or control siRNA (blue lines). The data are plotted relative to maximal levels with the control siRNA (taken as 100) and are presented as means ± SEM from the average of two biological replicates (n = 2). (F and G) The change in the ratio of GFP negative to GFP positive *Rex1*GFPd2 cells 28 hrs after 2i withdrawal in the presence of the siRNAs against the indicated *dusp*s relative to control siRNAs is shown. Data are the average of two biological replicates. (H) RT-qPCR analysis of the changes in *nanog* mRNA expression in *Rex1*GFPd2 cells upon 2i withdrawal for 28 hrs and depletion of the indicated *dusp*s. The data are normalised by the average of three reference genes, and presented relative to control siRNAs. Data are presented as means ± SEM and are the average of two biological replicates (n = 2). (I) RT-qPCR analysis of the expression of the indicated lineage marker genes following treatment of cells with siRNAs against *dusp1* or *dusp6* or a non-targeting control (ctrl) and release from “2i” for 3 (top) or 5 days (bottom). Data are presented as means ± SEM (n = 2). (J) Summary diagram illustrating the key regulatory role of the Dusps in mediating the action of the ERK pathway in early cell fate decisions during loss of pluripotency and onset of differentiation.

First we examined the set of genes we identified which accelerated differentiation in our primary siRNA screen for candidate *dusp* genes as we expected the loss of DUSPs would be predicted to enhance ERK phosphorylation and promote exit from pluripotency. *Dusp1*, *dusp3* and *dusp15* were amongst this category of genes (Table S2). We therefore determined the expression of these genes and a range of additional phosphatases in embryonic stem cells before and after “2i” removal. Amongst the genes tested, *dusp1*, *dusp5* and *dusp6* levels all increased following “2i” withdrawal while *dusp14* levels were fairly constant (Figure 5A; Figure S13A). The increased expression of all these phosphatases was dependent on active ERK pathway signalling as expected from other cellular systems (reviewed in [39]) but in the case of *dusp1* combinatorial inhibition of ERK and GSK signalling was required for maximal inhibition (Figure 5B; Figure S13B). However, at the protein level, Dusp1 levels gradually declined following “2i” withdrawal while Dusp6 levels increased in line with the increases in their mRNA levels (Figure S13C). Due to their dynamic expression, we focussed on Dusp1, Dusp5 and Dusp6 as these have the potential for controlling ERK pathway activity during embryonic stem cell differentiation. We therefore asked whether depletion of any of the genes identified in our screen would affect Dusp1, Dusp5 and Dusp6 expression at the mRNA or protein levels. Almost all the siRNAs tested (9/10) caused an increase in basal *dusp1* mRNA levels and the same was observed on *dusp6* levels for 4/10 genes (Figure 5C). In contrast, none of the siRNAs caused increases in *dusp5* levels under these conditions (Figure S13D). Similarly, the levels of these *dusps* followed a similar pattern in response to siRNA treatment after release from “2i” for 40 mins (Figure S13E). Importantly, increases in Dusp1 and Dusp6 at the protein level were also observed which generally correlated with the effects of these siRNAs on mRNA levels (Figure S13F) although there were exceptions typified by Rab24 whose depletion does not affect *dusp1* mRNA levels but instead appears to act post-transcriptionally to cause increased levels of Dusp1 protein.

An increase in the basal levels of MAP kinase phosphatases would likely lead to changes in the ERK activation kinetics, leading to the decreases in phosphorylated ERK levels we observed previously (Figure 4A). Indeed all of the siRNAs tested which promote increases in Dusp levels also cause a delay in peak activation of ERK and a subsequent reduction in the magnitude of this activation (Figure 5D; Figure S13G). Importantly other control siRNAs do not elicit this effect (Figure S14A). This suggests a causative link between the genes we identified in our screen, their effects on *dusp* gene expression and subsequent changes in ERK activity and downstream differentiation. Two key predictions of this model are that reductions in Dusp levels should first increase the rate and level of ERK pathway activation, and secondly, promote differentiation of embryonic stem cells. Indeed, depletion of Dusp6 and Dusp1 levels caused premature and higher amplitude activation of ERK whereas depletion of Dusp3 and a range of other Dusps had little effect on ERK activity levels (Figure 5E; Figure S14A). Importantly, while depletion of *dusp1* and *dusp6* caused increased levels of ERK activation, no increases could be detected on the low levels of Jnk and p38 phosphorylation, demonstrating a specific effect on the ERK pathway (Figure S14B). In our primary siRNA screen, we found that *dusp1* depletion enhanced the loss of *rex1* promoter-driven GFP expression (Figure 5F). We therefore depleted other Dusps to examine whether might function in an analogous manner and found that amongst these, only reductions in *dusp6* levels triggered more efficient inactivation of the *rex1-GFP* reporter gene (Figure 5G). Similarly, *dusp1* and *dusp6* depletion caused increased loss of mRNA expression of the pluripotency marker *nanog* whereas *dusp3* depletion had little effect (Figure 5H). Thus Dusp1 and Dusp6 appear to play an important role in maintaining pluripotency.

We extended this analysis to examine whether depletion of *dusp1* or *dusp6* affected lineage commitment by examining the expression of different marker genes 5 days after “2i” withdrawal. The depletion of *dusp1* caused increased expression of all three lineage markers, consistent with a general role for this gene in inhibiting loss of pluripotency (Figure 5I). In contrast, depletion of *dusp6* only caused increased levels of the ectoderm marker *nestin*, suggesting a more specific role in controlling differentiation into this lineage (Figure 5I).

Together, these results therefore demonstrate that our RNAi screen has enabled us to identify an important role for a subset of MAP kinase phosphatases in determining the rate and efficiency of ERK pathway activation in embryonic stem cells, and hence influence their ability to escape from pluripotency and begin to differentiate.

## Discussion

The derivation of pluripotent iPS cells and the controlled differentiation of embryonic stem cells into defined cell fates are two of the most important areas of research in the area of regenerative medicine. Numerous studies have helped build up a view of the complex signaling and transcriptional networks involved in maintaining the pluripotent state of embryonic stem cells (reviewed in [2]–[3]) but in contrast, much less is known about the pathways leading to the loss of pluripotency. Here we have conducted a genome-wide siRNA screen and identified over 400 genes which play a role in the onset of differentiation which allows ES cells to initiate escape from pluripotency. The vast majority of these genes have not previously been implicated in this process. This dataset therefore provides an important resource for the community and is a rich source of information for further investigating this phenomenon and also for a more basic understanding of the mechanisms governing the regulation and action of the core ERK and GSK3 signaling pathways.

Due to the controlled conditions used in our screen, we were able to link the genes which we identified to either the ERK and/or the GSK3 pathways as potential regulators or mediators of pathway functions. Importantly, it appears likely that many genes we have identified might also be important in the context of different culture conditions such as the commonly used serum and LIF-containing media (see Figure S12). However, further analysis on a case by case basis is required to substantiate a role for individual genes under these conditions. It is important to emphasise that ES cells grown in LIF and “2i” conditions exhibit very different epigenetic landscapes, so only a partial overlap in regulatory factors is expected when comparing these conditions [40]. Indeed, this is not unexpected considering that RNAi screens, including our own, commonly identify chromatin and transcriptional regulators as major important functionally enriched categories (see Figure 2E). Here we focused on genetic interactions with the ERK pathway, and we were able to place a large number of genes as acting upstream or downstream from ERK (Figure 4). Further subpartitioning of the dataset enabled us to identify genes which functioned upstream of Ras or between Ras and ERK (Figure 4D; Table S6). A surprising finding was that all of the genes which acted downstream of Ras, controlled ERK activation levels through controlling the levels of the MAP kinase phosphatases Dusp1 and/or Dusp6. The major point of control was at the transcriptional level. MAP kinase phosphatases are known regulators of MAP kinases activity in different cellular contexts, and Dusp6 in particular operates as part of a feedback loop in response to ERK activation (reviewed in [39]). While Dusp6 is able to specifically dephosphorylate and inactivate ERK *in vitro*, Dusp1 can also target the stress activated MAP kinases, JNK and p38 (reviewed in [39]). However, we saw no evidence for elevated levels of phosphorylated Jnk and p38 in mouse embryonic stem cells upon depletion of Dusp1, indicating its effects are likely via ERK. Fluctuations in both Dusp1 and Dusp6 levels occur upon ERK pathway activation in ES cells, suggesting that they play an important feedback regulatory role in this system. It appears likely that the combined amounts of these phosphatases helps set the threshold for ERK activation and hence ERK-mediated loss of pluripotency (Figure 5J). Indeed, tampering with this threshold control switch, either by depleting genes that control Dusp levels, or by directly depleting *dusp1* or *dusp6*, alters this threshold and changes the activation kinetics of the ERK pathway. This in turn accelerates the loss of pluripotency and increases the expression of lineage-specific markers, indicating that Dusps help control the equilibrium between pluripotency and differentiation by maintaining the correct levels of ERK activity. Our demonstration of a key role for Dusps in early ES cell differentiation, adds to the literature demonstrating the role of these enzymes in controlling developmental processes (reviewed in [41]) and illustrates the importance of establishing signaling thresholds by balancing activating and inactivating mechanisms which converge on ERK pathway signaling. Indeed, a recent study demonstrated a role for a different phosphatase, Dusp9, in maintaining pluripotency in mouse embryonic stem cells maintained in the presence of LIF and BMP4 [42]. In this study, BMP4 was implicated in upregulating *dusp9* expression through Smad pathway activation and hence leading to a dampening down of ERK activity. Importantly, they demonstrated that Dusp9 was not relevant to ERK control in stem cells maintained in 2i conditions, and rather as we have demonstrated, Dusp1 and Dusp6 are more important under these conditions. Reciprocally, we have shown that depletion of either *dusp1* or *dusp6* does not affect escape from pluripotency in ES cells released from maintenance in serum plus LIF conditions (Figure S12). Together these studies emphasise the critical importance of Dusps in controlling ERK signaling levels in stem cells to regulate the decisions about escape from pluripotency. Importantly, two of the top hits we identified in our screen, *gmnn* and *3830406c13rik*, which act to control Dusp levels and hence ERK activation kinetics, are not only involved in the loss of pluripotency but also in the appearance of differentiation markers for all three lineages (Figure 3). At this stage, it is unclear how these proteins impact on ERK pathway regulation at the molecular level but it points to a pivotal role of these proteins in controlling this key cellular fate decision.

In addition to regulating ERK activation, it is clear that many of the genes identified in our screen contribute to other molecular and biological processes. For example, there are a large numbers of genes encoding transcription and chromatin regulators identified (Figure 2E; Figure S6). This is not unexpected as cells must make wholesale changes in their gene expression programmes as they lose pluripotency and begin to differentiate (reviewed in [43]). There is also enrichment in our screen of functional categories of genes associated with core cellular metabolism and cell cycle control, which presumably reflects the changing anabolic, catabolic and proliferative requirements of the cells as they receive altered signaling input which might contribute to their change in identity (Figure 2E). In addition to enrichments of specific functional categories of genes, many of the genes show strong interconnectivities, implying that we have also uncovered functionally interdependent networks of genes which are important in specifying stem cell fate. This is particularly apparent amongst cell cycle regulators, transcription factors and chromatin modifiers where functionally distinct subnetworks can be observed but also clear interactions between the different subnetworks are apparent. Future studies are required to probe the functional relevance of the networks we have uncovered.

One of the future challenges will be to connect the genes identified in our screen with the ERK and GSK3 signalling pathways. We have begun to do this by focusing on a subset of genes associated with the ERK pathway. However, even though we have implicated many genes in controlling ERK activity, the only information we have for 35 of these genes, is that their point of action is downstream from ERK activation. ERK signaling might be needed to activate their expression (either directly or indirectly) or alternatively the genes might encode proteins which are directly phosphorylated by ERK. For example, it is known that transcriptional regulators such as Ets1, Jun and FoxO1 can all be phosphorylated by ERK in other situations [44]–[46]. More complicated mechanisms can also be envisaged where, for example, ERK and/or GSK3 signalling might converge on the activation of a key target gene, in parallel to one of the other regulators identified in this screen. Additional methodologies will need to be applied to help provide these links.

Another key issue to address is whether the ERK and GSK3 pathways work together or in parallel manner, to target different substrates and ultimately control different gene expression programmes and biological functions in ES cells. The two pathway inhibitors have both distinct and overlapping affects on ES cells (reviewed in [11]). Consistent with this, our study suggests that there may well be specific biological functions associated with GSK3 and ERK pathway signaling as different GO terms are enriched in hits from our screen. However, for the most part, the GO terms are often shared by genes associated with both pathways (see Figure 2 and Figure S6), suggesting that there might be a high degree of cooperativity. Indeed, it is well established that ERK-dependent phosphorylation often acts as a priming event for GSK3-mediated phosphorylation of substrates as exemplified by Smad1 [47]. Thus it appears likely that the pathways might act more generally in a combinatorial manner, either at the level of phosphorylation of common substrates or through convergence in activating gene expression through targeting distinct regulatory factors.

In summary, this study has identified an important role for the precise modulation of ERK MAP kinase signaling levels in the ability of a cell to exit the pluripotent ground state. Furthermore, we have identified a large number of genes that potentially impact on the function of the ERK pathway and GSK3 function in embryonic stem cells. It is becoming increasingly obvious that modulating these pathways has a potential impact on the reprogramming of somatic cells to the iPS cell state (reviewed in [3]) and reciprocally in promoting the differentiation of ES and iPS cells down defined lineages. Thus, the resource we have generated has paved the way for designing alternative strategies to either promote pluripotency or the subsequent generation of new cell identities for therapeutic purposes.

## Materials and Methods

### Tissue culture, RNA interference, and RT–PCR

ES cells were generally maintained in NDiff N2B27 media (Stem Cells, Inc.; scs-sf-nb-02) in the presence of the GSK3 inhibitor CHIR99021 (Stemgent, 04-0004; 3 µM) and MEK inhibitor PD0325901 (Stemgent, 04-0006; 1 µM) (“+2i” media) and were routinely passaged using Accutase (Sigma, A6964) every other day. For differentiation, the media containing inhibitor was removed and replaced with NDiff N2B27 media. Where indicated, ES cells were maintained in serum/LIF conditions in media containing knockout DMEM (Invitrogen 10829-018), 15% heat inactivated FBS (Invitrogen 10082-147), 2 mM of Glutamax-1 supplement (Invitrogen 35050-038), 1% non-essential amino acids (Invitrogen 11140-035), 50 µM 2-mercaptoethanol (Invitrogen 31350-010) and 5×10^5^ U of LIF (Millipore 10^3^ U/ml). The ES cells cultured under “+2i” conditions were adapted in serum/LIF culture conditions for at least 8 passages before the experiments were performed. Cells were stained for alkaline phosphatase expression using an alkaline phosphatase detection kit as described by the manufacturer's (Millipore).

For RNAi, 4×10^4^/cm^2^ cells (ie 1.28×10^4^ cells/well of 96 well plate) were plated out into a mixture of 0.3 µl of RNAi Max (Invitrogen) and 100 nM siRNA in 100 µl of “+2i” media for 24 hrs. All validation experiments used ON-TARGETplus siRNA SMART pools from Dharmacon.

Real time RT-qPCR was carried out as described previously [48]. For assays in 96 well plate format, the same basic protocol was followed except the RNA was obtained using a Fastlane cell RT-PCR kit (QIAGEN). Data were normalized for the average expression of the control genes *gapdh*, *hmbs* and *tbp*. The primer-pairs used for RT-PCR experiments are listed in Table S7.

### Western blot analysis

Western blotting was carried out with the primary antibodies; Erk2 (137F5; Cell Signalling, 4695), phospho-ERK (E10; Cell Signalling, 9106), Dusp1 (MKP-1; Upstate, 07535), Dusp6 (MKP-3; Epitomics, 2138-1) and Pou5f1 (Oct-3/4; Santa Cruz, sc-8628). All experiments were carried out in 96-well plates. The lysates were directly harvested in the 2×SDS sample buffer followed by sonication (Bioruptor, Diagenode). The proteins were detected using infrared dye-conjugated secondary antibodies (LI-COR Bioscience, IRDye 800CW [1 in 10,000] and IRDye 680LT [1 in 20,000]), and the signal was collected with a LI-COR Odyssey Infrared Imager and quantified using Odyssey software (LI-COR Bioscience, Odyssey Infrared Imaging system application software version 3.0.25).

### Ras activation assay

The Ras activities were examined using Ras activation ELISA assay kit (Millipore) as described in the manufacturers' instructions. The total lysates used in the ELISA assay was normalised with the quantity of the proteins assayed by the BCA protein assay kit (ThermoScientific).

### Flow cytometry analysis

Flow cytometric analysis was carried out using a LSRII flow cytometer and samples were loaded using HTS loader (BD Biosciences). For sampling, media was removed from each well. Single cell suspensions were generated by treating cells with accutase at 37°C for 7 mins followed by resuspendion in 0.03% BSA/PBS. Dead cells were stained by Sytox Red dead cell stain (Invitrogen, 5 nM). The cells were analysed immediately after sampling. Each sample was analysed with 10,000 event counts with the flow rate at 1 µl/s. The resulting GFP profile (green channel) was created by gating with the right ranges of cell sizes based on forward and reverse scatter plot (ssc vs fsc; blue channel) and dead cells were gated away based on the Sytox Red stain profile (red [APC] channel).

### siRNA library screening

All liquid handling processes were performed using Biomek robotic system (Beckman Coulter). For the primary screen, *Rex1*GFPd2 ES were grown in 96 well plates in the presence of “2i” and reverse transfection was performed using siGENOME siRNA pools (Dharmacon; mouse protein kinase [G-013500], GPCR [G-013600], druggable [G-014600] and genome [G-015000] libraries). 24 hrs later, the “2i” media was removed and replaced with fresh NDiff N2 B27 media. After 28 hrs, the levels of GFP in the cells were determined by flow cytometry as described above. Each plate contained 8 control non-targeting siRNAs, and the positive control siRNAs against *gsk3β* and *fgf4*. To take into account slight variations in the timing of pluripotency loss, the ratio of high GFP to low GFP expressing cells was established on each plate based on the non-targeting controls, allowing a threshold to be set as 1 (ie 50% high GFP and 50% low GFP). This threshold was used to determine the ratio of high to low GFP expressing cells in the other wells. The mean plus/minus standard deviation (SD) was calculated for each plate, and individual wells were scored positive if they exceeded 2×SD above or below this mean. The screen was performed in duplicate, with duplicate plates being analysed on different days. A final list of positive hits was determined by taking siRNAs which scored an average of 2×SD across both plates (or on a single plate where the duplicate well was defective in the case of 30 siRNAs), generally with both plates scoring >1.5×SD above the mean. However, an additional small number of siRNAs were scored as positive where the average score was >2.5×SD above the mean where only one plate had to score >1.5×SD above the mean, and also for seven siRNAs where the average score was >1.9×SD above the mean and both plates scored >1.9×SD above the mean.

For the validation screens, either *Rex1*GFPd2 or *Oct4*GFP ES cells were used and screens were performed as above except that ON-TARGETplus siRNA duplexes were used and GFP levels in *Oct4*GFP ES cells were determined 72 hrs after release from “2i”. Individual wells in each screen were scored as positive if the average GFP(+)/GFP(−) ratio exceeded 1.25×SD above the non-targeting controls across both duplicate plates. Additional hits were considered as positive if they scored >0.8×SD above the mean in one validation screen and also scored >1.0×SD above the mean in the other.

For the “1i” screens, *Rex1*GFPd2 were used as for the validation screens but only one inhibitor (ie either CHIR99021 or PD0325901) was withdrawn. Wells were scored as positive if the average GFP(+)/GFP(−) ratio exceeded 1.5×SD above the mean of the non-targeting controls.

### Bioinformatics analysis

For constructing networks, lists of gene names were uploaded into STRING [49] with the confidence score set high (0.40). The resulting networks were saved as *.txt files and then uploaded into Cytoscape (v. 2.7.0) choosing coexpression, textmining, knowledge and experimental data as proximity criteria. yFiles→organic network layouts were applied and the positioning and graphic representation of nodes were adjusted manually for increased clarity.

GO term analysis was carried out using DAVID Bioinformatics Resources 6.7 (NIH) [50]. The enriched terms from the functional annotation chart were extracted and manually clustered. Heat maps of GO terms were generated by MultiexperimentViewer (MeV 4_7_4). GO term summary and visualization was carried out by REVIGO [51].

## Supporting Information

Figure S1 Kinetics of GFP expression level changes in the primary and validation library screens. (A and B) FACS profiles of GFP expression in *Rex1*GFPd2 ES cells grown for the indicated times in the presence of the inhibitors CHIR99021 and PD0325901 (“2i”) or upon removal of the inhibitors (“−2i”). The profiles corresponding to high GFP expressing cells are shown in dark green in (A). The effect of pre-treating cells with control non-targeting siRNAs (green; ctrl) or siRNAs against *fgf4* (blue) or *gsk3* (red) on the GFP expression profile is shown in (B). The dashed line shows the position of the centre of the peak of the starting population of cells. (C) FACS profiles of GFP expression in *Oct4*GFP ES cells grown for the indicated times following removal of the inhibitors (“−2i”) in the presence of the indicated siRNA constructs (as labelled in B). Clear shifts in the distributions of high versus low GFP expressing cells can be observed between 27 and 30 hr for the *Rex1*GFPd2 ES cells and at 72 hrs for the *Oct4*GFP cells after removal of “2i” in the presence of *fgf4* or *gsk3* siRNAs.
(PDF)

Figure S2 Representative FACS profiles in each of the library screens. (A–D) FACS profiles of GFP expression in the presence of control non-targeting siRNAs or siRNAs against the indicated genes. The numbers next to each gene name indicate plate and well numbers and the numbers above each graph are the corresponding GFP high/GFP low ratios. Example profiles from the following screens are provided; column 1, primary “2i” screen using *Rex1*GFPd2 ES cells; column 2, validation “2i” screen using *Rex1*GFPd2 ES cells; column 3, validation “2i” screen using *Oct4*GFP ES cells; column 4, secondary “1i” screen using *Rex1*GFPd2 ES cells upon removal of CHIR99021; column 5 secondary “1i” screen using *Rex1*GFPd2 ES cells upon removal of PD0325901. Examples are shown for siRNAs which affect the ratio of high/low GFP expression (A) specifically when only the ERK pathway inhibitor is removed (ERK only), (B) only the GSK3 inhibitor is removed (GSK only), (C) either the ERK pathway or GSK inhibitors are removed (ERK/GSK) or (D) only when both the ERK pathway and GSK inhibitors are removed (ERK & GSK). (E) Example FACS profiles of GFP expression in the presence of control non-targeting siRNAs or siRNAs against the indicated genes from the primary “2i” screen, for hits which demonstrate enhanced loss of GFP expression. .
(PDF)

Figure S3 Enriched functional categories of genes in the high confidence dataset. Enriched classes of genes amongst the 292 high confidence hits resulting from the validation screens [depicted in (A)] were determined by searching for Gene Ontology (GO) terms using DAVID. (B, D and G) Data are shown graphically according to their relative P-values or (C, E and H) data are visualized using REVIGO. GO terms are grouped according to the level of complexity of the terms; (B and C) molecular function, (D and E) biological process and (G and H) cellular component. (C, E and H) data are plotted according to the size of the GO term category (y-axis; also reflected in the size of the circles) and the significance of the association with the category (x-axis). The identities of the most significant categories are indicated. (F and I) Analysis of interacting networks of enriched GO terms from the molecular function (F) and the cellular component (I) categories, depicted using the “interactive graph” view of REVIGO.
(PDF)

Figure S4 Kinetics of GFP expression level changes in the “1i” screens. GFP expression profiles of *Rex1*GFPd2 ES cells grown for the indicated times in the presence of the inhibitors PD0325901 and CHIR99021 (“2i”), upon removal of both of the inhibitors (“−2i”), or removal of either the PD0325901 or the CHIR99021 inhibitors (“−1i”). The data are shown graphically (A) and as FACS profiles of GFP expression 60 hrs after inhibitor removal (B).
(PDF)

Figure S5 Heatmap summary of the hits from the “1i” screens. The heatmap summary depicts the stratification of the hits according to their effects on the GFP(+)/GFP(−) ratio upon withdrawal of the GSK3 inhibitor (GSK3) or MEK inhibitor (ERK). Red indicates an increased ratio and blue represents a decreased ratio, and intermediate colours given according to the scale bar. Hits are grouped as specific to the ERK or GSK3 pathways (left side map), related to either the ERK or the GSK3 pathways (central map; ERK/GSK3) or only functional when both pathways are active (right side map; GSK3 and ERK)
(PDF)

Figure S6 Heatmaps of the enriched GO terms identified for genes corresponding to high confidence hits from the validation screens. The heatmap distribution of the full list of significant GO terms identified specifically associated with the ERK (133), or GSK3 (168) pathways and the total 274 validated hits. Each GO term is scored by −log_10_(P-value), and coloured according to the bar shown below the figure. Terms corresponding to distinct functional groups are manually clustered (indicated on the left), and are then ranked according to their significance scores in the “ERK only” dataset.
(PDF)

Figure S7 Heatmaps of the specific subsets of enriched GO terms identified for genes corresponding to high confidence hits from the validation screens. Heatmap distributions of specific subsets of significant GO terms identified specifically associated with the categories of hits described in Figure S6; (A) Cancer pathways, (B) Cell cycle terms, (C) Mitochondrial terms, (D) Developmental processes. The associated GO term descriptions are indicated on the right (the GO terms enriched in only the ERK or GSK3 categories are indicated in red or green font respectively).
(PDF)

Figure S8 Interaction networks of genes associated with high confidence hits from the validation and secondary screens. STRING network analysis of the core network formed by the 274 genes associated with signal-dependent loss of pluripotency and promoting early differentiation processes in the mouse embryonic stem cells. Genes are grouped according to common biological processes. (A) In addition to a highly connected central network (left side; see Figure 2E), there are also two subnetworks with no known connections to this central nexus which are both associated with aspects of RNA processing (right side). Each gene is colour-coded according to the pathway(s) it is associated with. (B) The network of the transcription and chromatin regulators identified in the screens. Factors are manually grouped according to the pathways they are directly associated with. Known links to the ERK (Nras-Raf1) and GSK3 (Gsk3b) pathway components identified in the screens are shown. The coloured lines of edges represent confidence scores of interconnectivity. Dark blue lines represent 0.8–1, light blue lines represent 0.6–0.8, and light grey lines represent 0.4–0.6 confidence levels, respectively.
(PDF)

Figure S9 The effects of depletion of genes identified in the validation screen on the expression of markers of pluripotency and early differentiation. (A) Venn diagram (top) illustrating the number and a list of selected screen hits from each category used in the subsequent studies. (B and C) RT-qPCR analysis of the effects of depletion of the indicated genes on the mRNA expression levels of the ES-specific genes, *nanog* and *rex-1* (B), and the early differentiation marker, *fgf5* (C). The expression levels were normalised by the average of three reference genes (refs; B) or *gapdh* (C). Data are shown for each siRNA duplex relative to the maximal expression exhibited in the presence of an siRNA pool (taken as 100). The blue dashed lines indicate the threshold level (>1.5 SD above (B) or below (C) the mean of the control [ctr] siRNAs). Genes which show changes exceeding (B) or below (C) this level, are indicated by red bars. Data are presented as means ± SEM and are the average of three biological replicates (n = 3). Heatmap summaries (see Figure 2B for details) are shown on the left illustrating the magnitude of the effects of the selected screen hits used in the subsequent studies, in each of the “1i” screens. These selected hits are distributed within three categories as indicated on the heatmap. (D) *rex1* (y-axis) and the reciprocal of *fgf5* (x-axis) mRNA expression levels upon 2i withdrawal for 36 hrs and 48 hrs, respectively, are plotted following knockdown of individual genes (see B and C for details). Data are shown for each siRNA duplex relative to the maximal expression exhibited in the presence of an siRNA pool (taken as 100). Dotted lines represent the expression values >2 standard deviations above the mean of the negative control siRNAs. Red dots represent siRNA duplexes which promote elevated expression of *nanog* and lower levels of *fgf5* (quadrant 1), whereas green (quadrant 3) and black (quadrant 4) dots represent siRNAs that cause changes at or below this threshold cut-off value for only one gene. The brown dot represents the negative control siRNAs. Blue dots (quadrant 2) represent siRNAs that cause elevated *nanog* expression but fail to show reductions in *fgf5* expression.
(PDF)

Figure S10 Western blot analysis of phospho-ERK levels upon depletion of genes identified in the secondary siRNA screens. ERK activation levels following 2i withdrawal (−2i) for 20 mins were determined by western blotting using a phospho-ERK-specific antibody (pERK; top panels) and normalized against total ERK levels (bottom panels). siRNAs against the indicated genes from the “1i” screens (ERK hits in black, GSK3 hits in brown and ERK/GSK3 hits in blue) or control non-targeting siRNAs (ctrl) are indicated. The presence or absence of inhibitors in the control samples is indicated (+/−2i). Dotted lines indicate where gels have been cut to remove irrelevant lanes and rejoined. The data shown are from one of three biological replicate experiments.
(PDF)

Figure S11 Summary of the effects of each screen hit on the activity of Ras and ERK. A heatmap summary (see Figure 2B for details) is shown on the left illustrating the magnitude of the effects of the selected screen hits in each of the “1i” screens. These selected hits are distributed within three categories as indicated on the heatmap. Genes are ranked according to their effect in the “1i” screen where only the MEK inhibitor was removed. The heatmap on the right illustrates the magnitude of effect of depletion of each of the indicated genes on the levels of phospho-ERK (pERK) or the activity of Ras (see Figure 4). The colour code shown below relates to the magnitude of change above or below the mean of the control siRNAs (calculated as number of standard deviations from the mean). Black lines indicate that the effect was not assayed in the Ras activation assay.
(PDF)

Figure S12 GFP expression level changes upon gene depletion under LIF conditions. FACS profiles of GFP expression in *Rex1*GFPd2 ES cells grown in the presence of LIF and released for 28 hrs. The profiles corresponding to high GFP expressing cells are shown in dark green. The effect of pre-treating cells with control non-targeting siRNAs or siRNAs against the indicated genes on the GFP expression profile under these conditions is shown. The ratio of high to low GFP expressing cells is shown quantitatively on the right as means ± SEM; n = 2–3). The vertical lines represent the mean of the non-targeting control (ctrl) (solid line) and two standard deviations above this mean (dotted line).
(PDF)

Figure S13 The regulation of Dusp activity and ES cell differentiation (A–B and D–E) RT-qPCR analysis of *dusp1*, *dusp5*, *dusp6* and *dusp14* mRNA expression in mouse ES cells. Data are normalised by the average values of three reference genes (ref) and are presented as means ± SEM and are the average of three biological replicates (n = 3). (A) The kinetics of *dusp5* and *dusp14* expression at the indicated times following 2i withdrawal. (B) The effects of the indicated inhibitors, either alone or in combination, on the expression of *dusp5* and *dusp14* at the indicated times following inhibitor withdrawal. (D and E) The effects of depletion of the indicated genes on *dusp5* mRNA expression in the presence, (D) or *dusp1*, *dusp5*, or *dusp5* mRNA expression in the absence, (E) of 2i. The blue dashed lines indicate the threshold level (2× SD above/below the mean of the negative controls) and levels below and above this are indicated by red and pink bars, respectively. The average activity in the presence of control siRNA (ctrl) is shown by the solid grey line. (C and F) Dusp1 and Dusp6 protein expression measured by western blot analysis (bottom panels) was quantified and normalized by Pou5F1 levels (shown graphically in the top panels). (C) The kinetics of Dusp1 and Dusp6 expression at the indicated times following 2i withdrawal. (F) The effects of depletion of the indicated genes on Dusp1 and Dusp6 expression in the presence of 2i. Data are the average of two experiments. (G) Active ERK levels were determined by the ratio of phospho-ERK (pERK)/total ERK (ERK) levels at the indicated times following 2i release for the indicated times in the presence of the indicated siRNAs (red lines) or control siRNA (blue lines). The data are plotted relative to maximal levels with the control siRNA (taken as 100) and are presented as means ± SEM from the average of two biological replicates (n = 2).
(PDF)

Figure S14 The role of Dusps in MAP kinase activation in ES cells. (A) Active ERK levels were determined by the ratio of phospho-ERK (pERK)/total ERK (ERK) levels at the indicated times following 2i release in the presence of the indicated siRNAs (red lines) or control siRNA (blue lines). The data are plotted relative to maximal levels with the control siRNA (taken as 100) and are presented as means ± SEM from the average of two biological replicates (n = 2). (B) Active levels of the indicated MAPKs were determined by the ratio of phosphorylated (p) to non-phosphorylated forms at the indicated times following 2i release in the presence of the indicated siRNAs (red lines) or control siRNA (blue lines). The data are plotted relative to basal levels with the control siRNA in the presence of 2i (taken as 1) and are presented as means ± SEM from the average of two biological replicates (n = 2).
(PDF)

Table S1 Positive hits identified in the primary and validation siRNA screens for genes involved in escape from pluripotency. (A) Positive hits from the primary siRNA screen in *Rex1*GFPd2 cells. (B) Positive hits from the validation screen in *Rex1*GFPd2 cells. (C) Positive hits from the validation screen in *Oct4*GFP cells. See Figure 1A for details.
(XLSX)

Table S2 Hits identified in the primary and validation siRNA screens for genes involved in maintaining pluripotency. Genes are listed when their depletion results in more loss of GFP-positive cells with both *Rex1*GFPd2 and *Oct4*GFP cells compared to negative controls. Hits were scored as positive according to the same criteria as those which scored positive for retention of pluripotency.
(XLSX)

Table S3 Overlaps with other siRNA screens. Overlaps between genes identified in this paper as important for promoting loss of pluripotency and onset of differentiation (Table S4) or the maintenance of pluripotency (Table S2) and previous genome-wide and focused siRNA screens conducted in mouse ES cells.
(PDF)

Table S4 Positive hits identified in both of the validation siRNA screens for genes involved in escape from pluripotency. These hits scored positive in validation screens with both *Rex1*GFPd2 and *Oct4*GFP cells and were considered as a high confidence group for further analysis.
(XLS)

Table S5 Hits eliminated in the counterscreen. Validated hits from the primary screens which also scored as positive in a screen with *Rex1*GFPd2 cells in the presence of the MEK and GSK3 inhibitors (“+2i”). These were not taken forward for further analysis due to the lack of a potential link to active ERK or GSK3 signalling.
(XLS)

Table S6 Summary of the point of action of siRNAs with respect to the ERK signaling pathway. Genes targeted by the siRNA constructs are grouped according to whether they act to potentiate from Ras activity, act downstream of Ras to activate ERK or play a role downstream from, or in parallel to, ERK signalling. Each column indicates the association of the genes with the ERK, GSK or both pathways, as determined by the “1i” screens (see Figure S5).
(PDF)

Table S7 PCR primers used in RT-qPCR. The sequences of both forward (F) and reverse (R) primers are provided.
(PDF)
