# Supplementary figures and images for: A Genome-Wide RNAi Screen Reveals MAP Kinase Phosphatases as Key ERK Pathway Regulators during Embryonic Stem Cell Differentiation
Source: PLoS Genet. 2012 Dec 13;8(12):e1003112. doi: 10.1371/journal.pgen.1003112 (PMC3521700; doi:10.1371/journal.pgen.1003112)

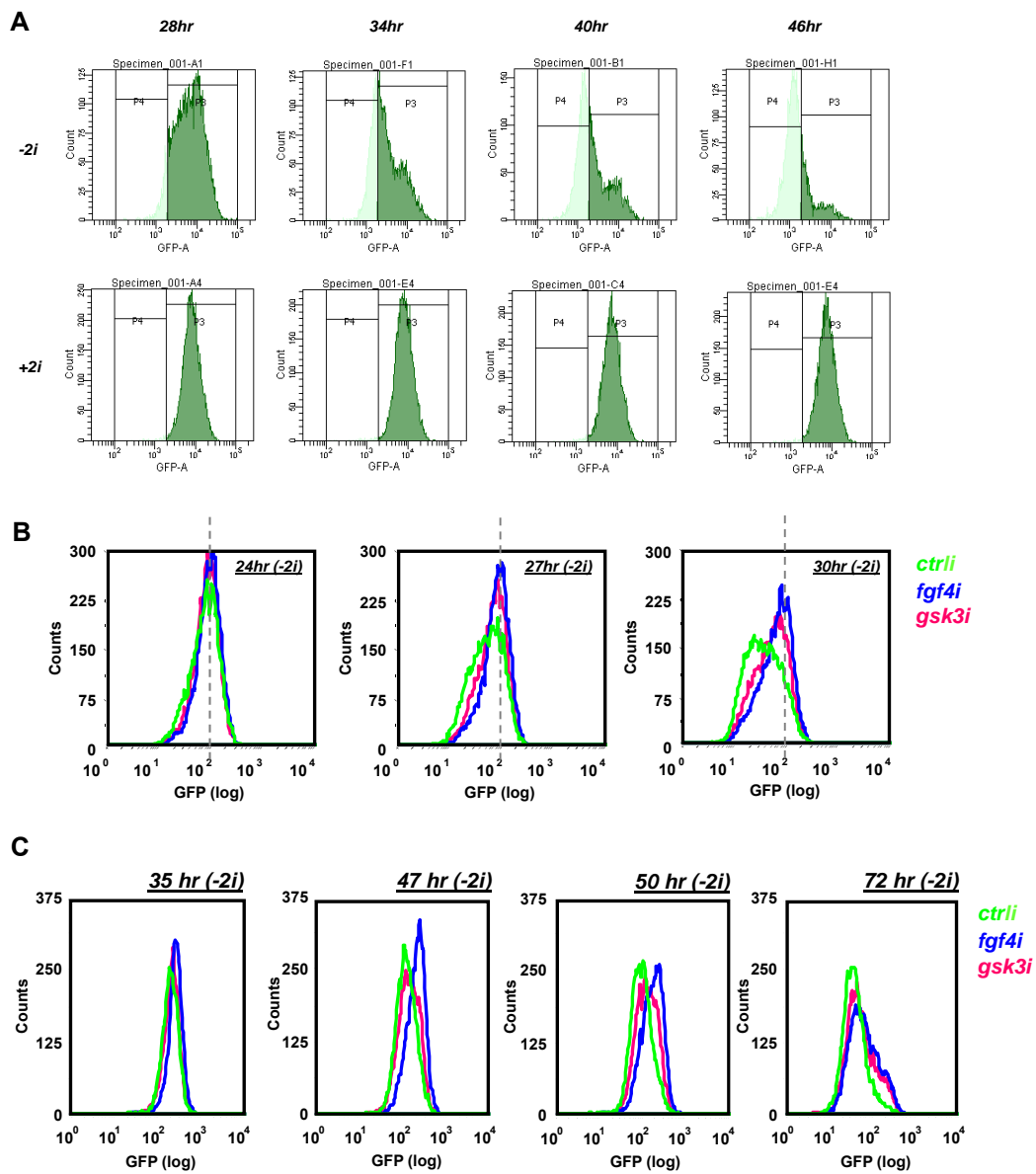

Supp. Fig. S1

Supplement: Figure S1 — Kinetics of GFP expression level changes in the primary and validation library screens. (A and B) FACS profiles of GFP expression in Rex1GFPd2 ES cells grown for the indicated times in the presence of the inhibitors CHIR99021 and PD0325901 (“2i”) or upon removal of the inhibitors (“−2i”). The profiles corresponding to high GFP expressing cells are shown in dark green in (A). The effect of pre-treating cells with control non-targeting siRNAs (green; ctrl) or siRNAs against fgf4 (blue) or gsk3 (red) on the GFP expression profile is shown in (B). The dashed line shows the position of the centre of the peak of the starting population of cells. (C) FACS profiles of GFP expression in Oct4GFP ES cells grown for the indicated times following removal of the inhibitors (“−2i”) in the presence of the indicated siRNA constructs (as labelled in B). Clear shifts in the distributions of high versus low GFP expressing cells can be observed between 27 and 30 hr for the Rex1GFPd2 ES cells and at 72 hrs for the Oct4GFP cells after removal of “2i” in the presence of fgf4 or gsk3 siRNAs. (PDF) [file pgen.1003112.s001.pdf]

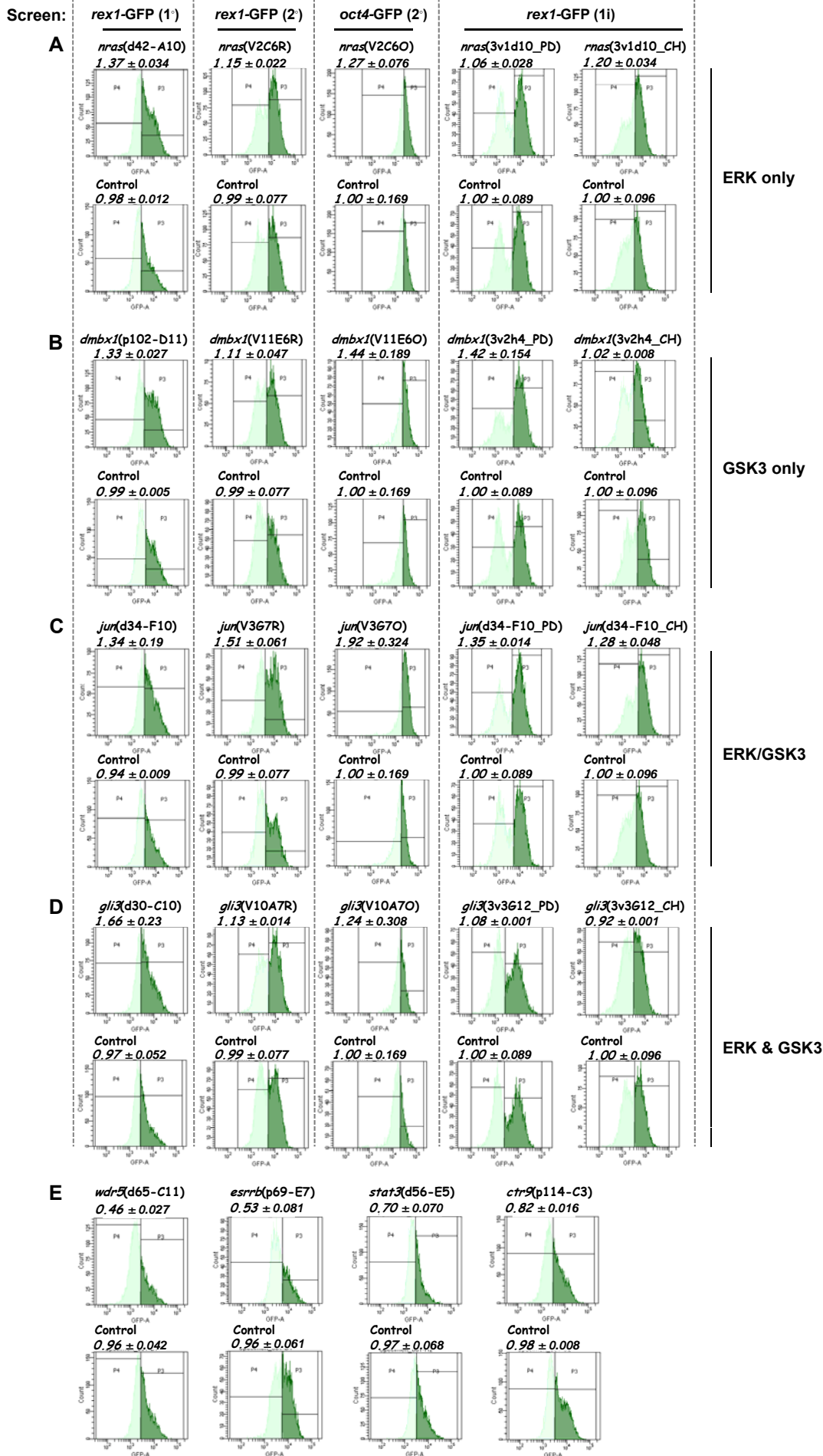

Supplement: Figure S2 — Representative FACS profiles in each of the library screens. (A–D) FACS profiles of GFP expression in the presence of control non-targeting siRNAs or siRNAs against the indicated genes. The numbers next to each gene name indicate plate and well numbers and the numbers above each graph are the corresponding GFP high/GFP low ratios. Example profiles from the following screens are provided; column 1, primary “2i” screen using Rex1GFPd2 ES cells; column 2, validation “2i” screen using Rex1GFPd2 ES cells; column 3, validation “2i” screen using Oct4GFP ES cells; column 4, secondary “1i” screen using Rex1GFPd2 ES cells upon removal of CHIR99021; column 5 secondary “1i” screen using Rex1GFPd2 ES cells upon removal of PD0325901. Examples are shown for siRNAs which affect the ratio of high/low GFP expression (A) specifically when only the ERK pathway inhibitor is removed (ERK only), (B) only the GSK3 inhibitor is removed (GSK only), (C) either the ERK pathway or GSK inhibitors are removed (ERK/GSK) or (D) only when both the ERK pathway and GSK inhibitors are removed (ERK & GSK). (E) Example FACS profiles of GFP expression in the presence of control non-targeting siRNAs or siRNAs against the indicated genes from the primary “2i” screen, for hits which demonstrate enhanced loss of GFP expression. . (PDF) [file pgen.1003112.s002.pdf]

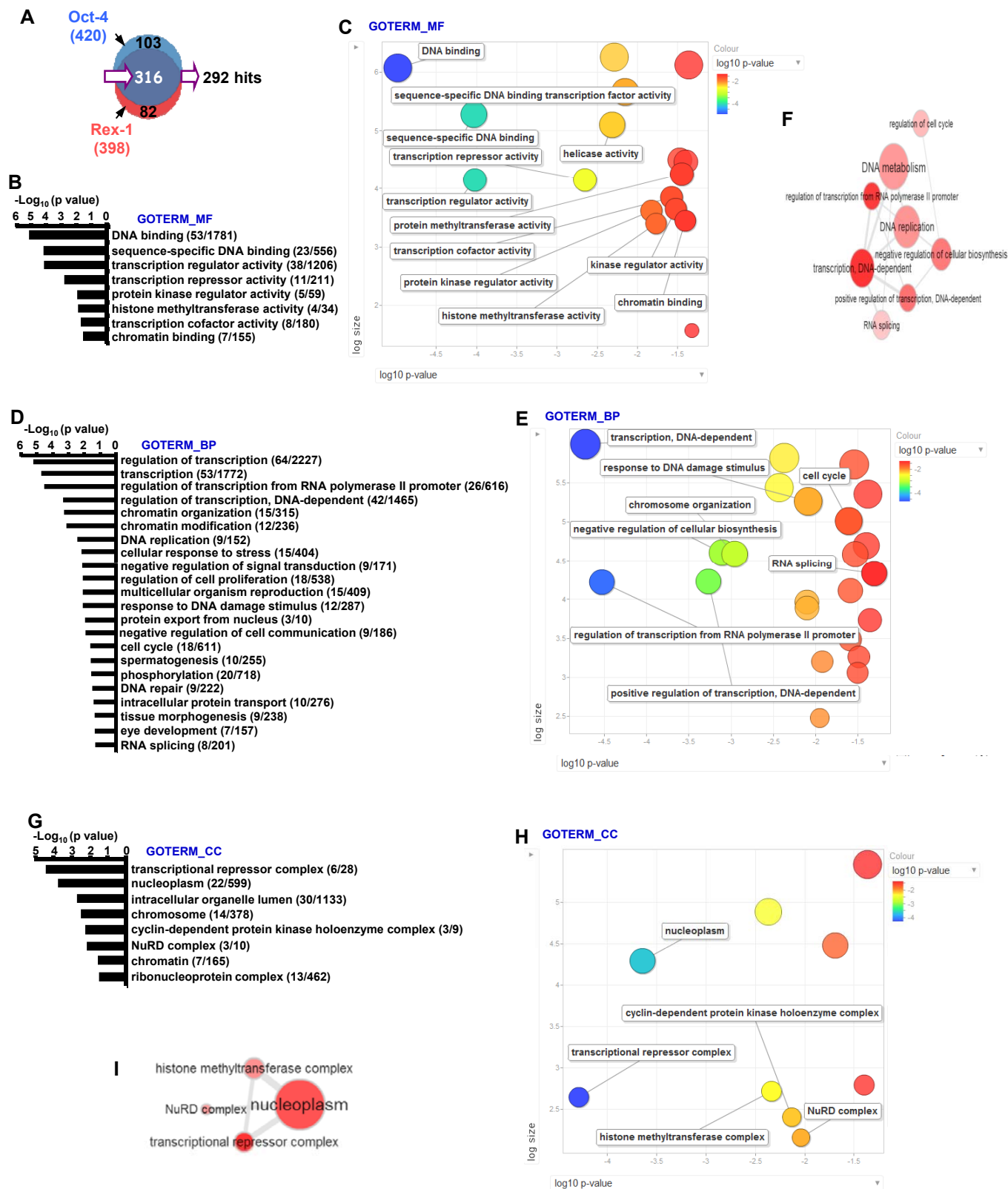

Supp. Fig. S3

Supplement: Figure S3 — Enriched functional categories of genes in the high confidence dataset. Enriched classes of genes amongst the 292 high confidence hits resulting from the validation screens [depicted in (A)] were determined by searching for Gene Ontology (GO) terms using DAVID. (B, D and G) Data are shown graphically according to their relative P-values or (C, E and H) data are visualized using REVIGO. GO terms are grouped according to the level of complexity of the terms; (B and C) molecular function, (D and E) biological process and (G and H) cellular component. (C, E and H) data are plotted according to the size of the GO term category (y-axis; also reflected in the size of the circles) and the significance of the association with the category (x-axis). The identities of the most significant categories are indicated. (F and I) Analysis of interacting networks of enriched GO terms from the molecular function (F) and the cellular component (I) categories, depicted using the “interactive graph” view of REVIGO. (PDF) [file pgen.1003112.s003.pdf]

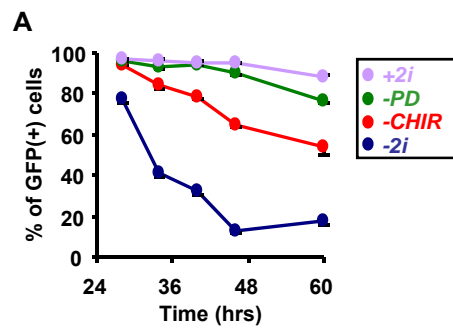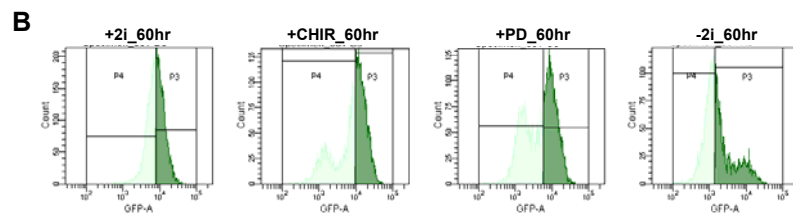

Supp. Fig. S4

Supplement: Figure S4 — Kinetics of GFP expression level changes in the “1i” screens. GFP expression profiles of Rex1GFPd2 ES cells grown for the indicated times in the presence of the inhibitors PD0325901 and CHIR99021 (“2i”), upon removal of both of the inhibitors (“−2i”), or removal of either the PD0325901 or the CHIR99021 inhibitors (“−1i”). The data are shown graphically (A) and as FACS profiles of GFP expression 60 hrs after inhibitor removal (B). (PDF) [file pgen.1003112.s004.pdf]

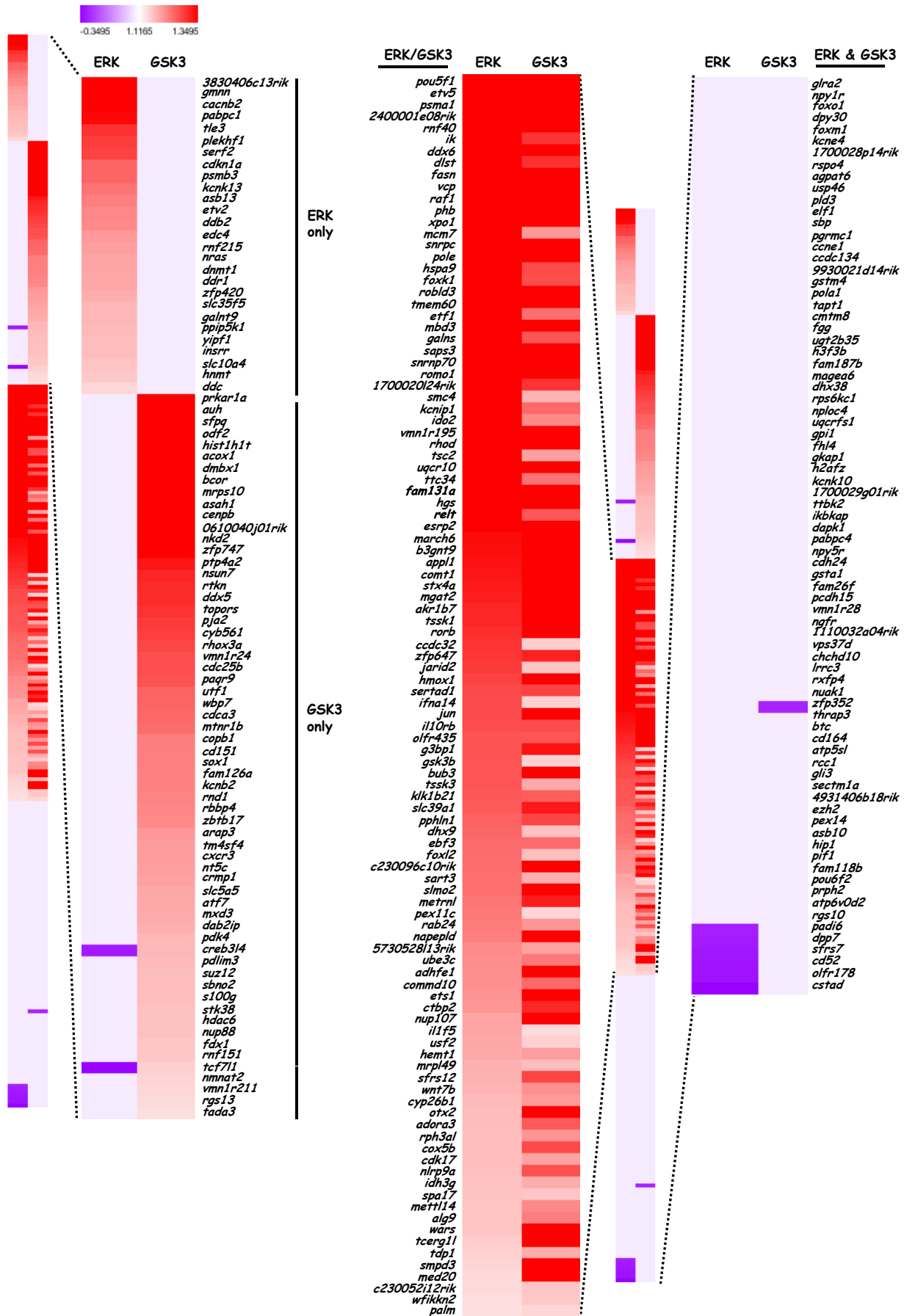

Supp. Fig. S5

Supplement: Figure S5 — Heatmap summary of the hits from the “1i” screens. The heatmap summary depicts the stratification of the hits according to their effects on the GFP(+)/GFP(−) ratio upon withdrawal of the GSK3 inhibitor (GSK3) or MEK inhibitor (ERK). Red indicates an increased ratio and blue represents a decreased ratio, and intermediate colours given according to the scale bar. Hits are grouped as specific to the ERK or GSK3 pathways (left side map), related to either the ERK or the GSK3 pathways (central map; ERK/GSK3) or only functional when both pathways are active (right side map; GSK3 and ERK) (PDF) [file pgen.1003112.s005.pdf]

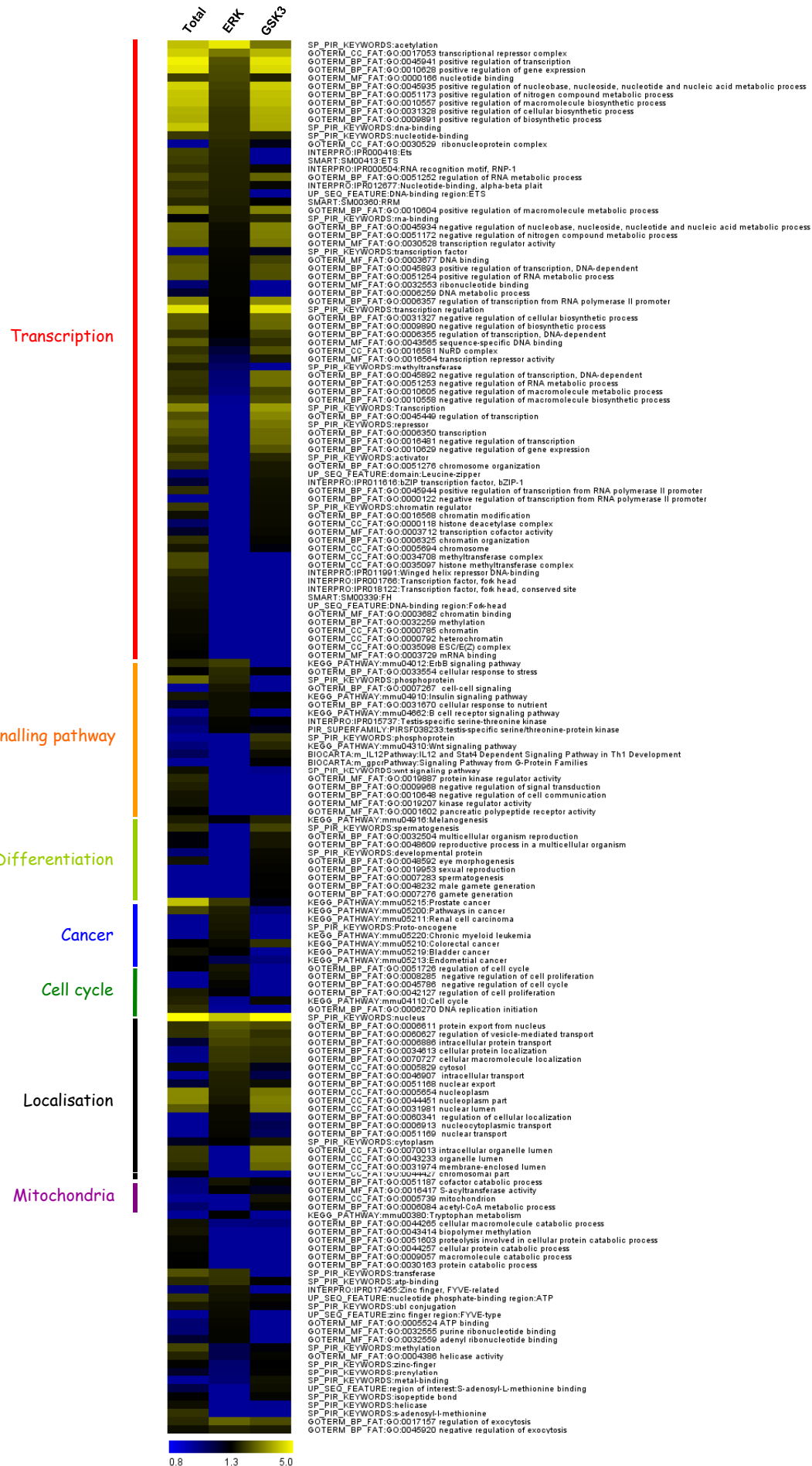

Supp. Fig. S6

Supplement: Figure S6 — Heatmaps of the enriched GO terms identified for genes corresponding to high confidence hits from the validation screens. The heatmap distribution of the full list of significant GO terms identified specifically associated with the ERK (133), or GSK3 (168) pathways and the total 274 validated hits. Each GO term is scored by −log10(P-value), and coloured according to the bar shown below the figure. Terms corresponding to distinct functional groups are manually clustered (indicated on the left), and are then ranked according to their significance scores in the “ERK only” dataset. (PDF) [file pgen.1003112.s006.pdf]

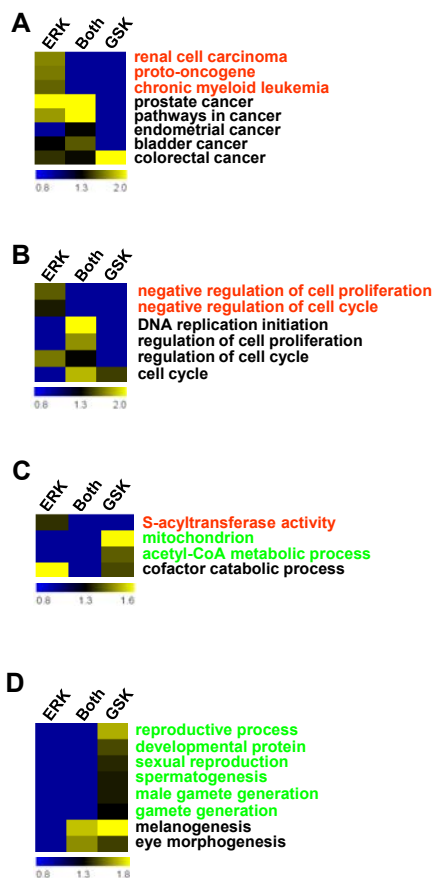

Supp. Fig. S7

Supplement: Figure S7 — Heatmaps of the specific subsets of enriched GO terms identified for genes corresponding to high confidence hits from the validation screens. Heatmap distributions of specific subsets of significant GO terms identified specifically associated with the categories of hits described in Figure S6; (A) Cancer pathways, (B) Cell cycle terms, (C) Mitochondrial terms, (D) Developmental processes. The associated GO term descriptions are indicated on the right (the GO terms enriched in only the ERK or GSK3 categories are indicated in red or green font respectively). (PDF) [file pgen.1003112.s007.pdf]

A

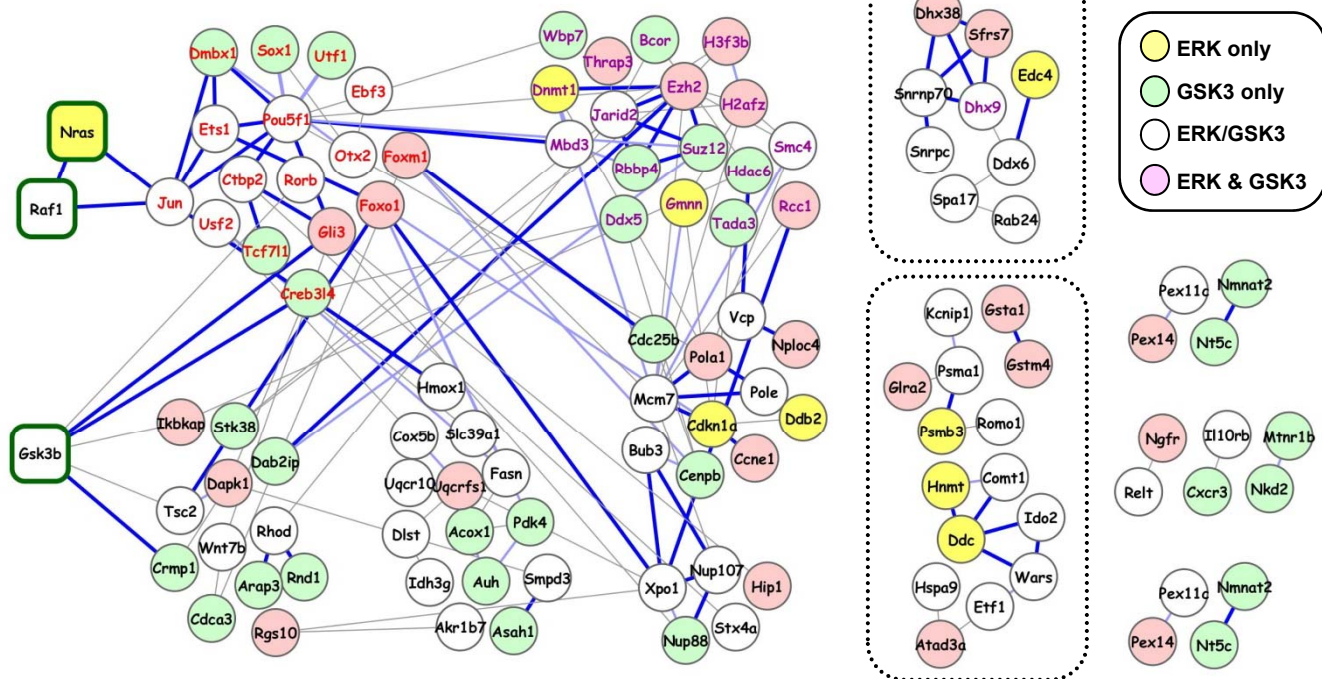

B

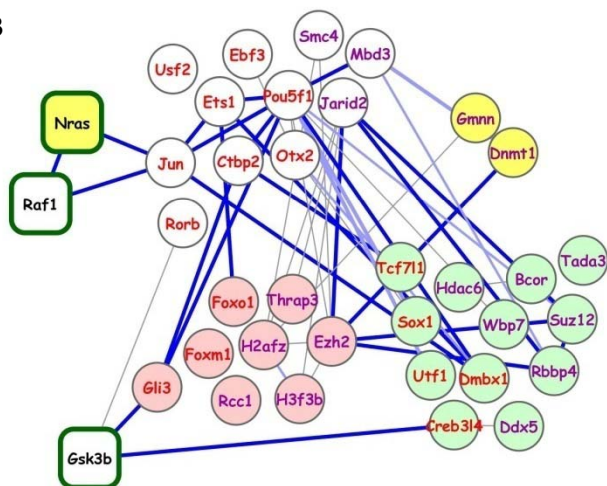

Supp. Fig. S8

Supplement: Figure S8 — Interaction networks of genes associated with high confidence hits from the validation and secondary screens. STRING network analysis of the core network formed by the 274 genes associated with signal-dependent loss of pluripotency and promoting early differentiation processes in the mouse embryonic stem cells. Genes are grouped according to common biological processes. (A) In addition to a highly connected central network (left side; see Figure 2E), there are also two subnetworks with no known connections to this central nexus which are both associated with aspects of RNA processing (right side). Each gene is colour-coded according to the pathway(s) it is associated with. (B) The network of the transcription and chromatin regulators identified in the screens. Factors are manually grouped according to the pathways they are directly associated with. Known links to the ERK (Nras-Raf1) and GSK3 (Gsk3b) pathway components identified in the screens are shown. The coloured lines of edges represent confidence scores of interconnectivity. Dark blue lines represent 0.8–1, light blue lines represent 0.6–0.8, and light grey lines represent 0.4–0.6 confidence levels, respectively. (PDF) [file pgen.1003112.s008.pdf]

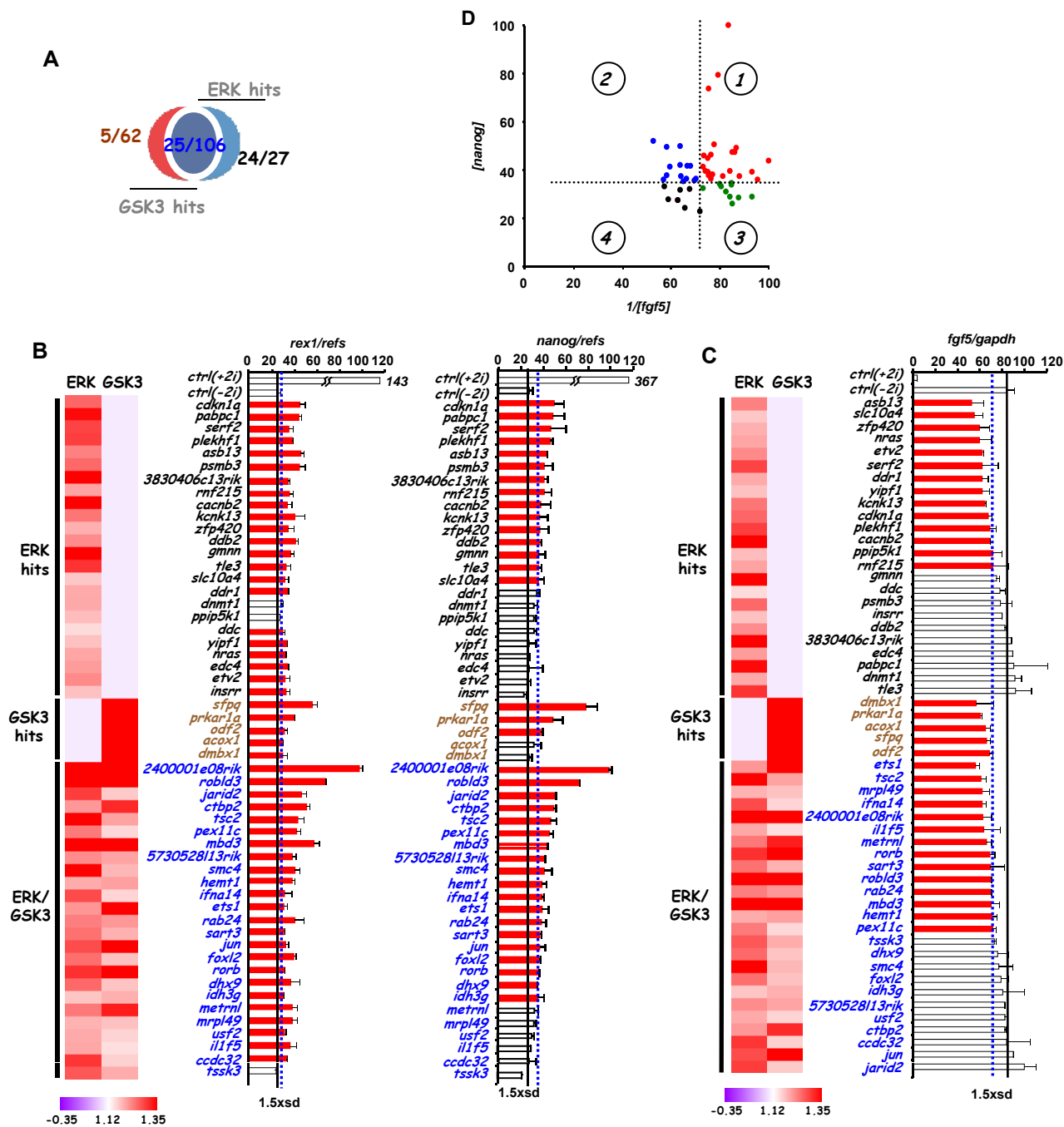

Supp. Fig. S9

Supplement: Figure S9 — The effects of depletion of genes identified in the validation screen on the expression of markers of pluripotency and early differentiation. (A) Venn diagram (top) illustrating the number and a list of selected screen hits from each category used in the subsequent studies. (B and C) RT-qPCR analysis of the effects of depletion of the indicated genes on the mRNA expression levels of the ES-specific genes, nanog and rex-1 (B), and the early differentiation marker, fgf5 (C). The expression levels were normalised by the average of three reference genes (refs; B) or gapdh (C). Data are shown for each siRNA duplex relative to the maximal expression exhibited in the presence of an siRNA pool (taken as 100). The blue dashed lines indicate the threshold level (>1.5 SD above (B) or below (C) the mean of the control [ctr] siRNAs). Genes which show changes exceeding (B) or below (C) this level, are indicated by red bars. Data are presented as means ± SEM and are the average of three biological replicates (n = 3). Heatmap summaries (see Figure 2B for details) are shown on the left illustrating the magnitude of the effects of the selected screen hits used in the subsequent studies, in each of the “1i” screens. These selected hits are distributed within three categories as indicated on the heatmap. (D) rex1 (y-axis) and the reciprocal of fgf5 (x-axis) mRNA expression levels upon 2i withdrawal for 36 hrs and 48 hrs, respectively, are plotted following knockdown of individual genes (see B and C for details). Data are shown for each siRNA duplex relative to the maximal expression exhibited in the presence of an siRNA pool (taken as 100). Dotted lines represent the expression values >2 standard deviations above the mean of the negative control siRNAs. Red dots represent siRNA duplexes which promote elevated expression of nanog and lower levels of fgf5 (quadrant 1), whereas green (quadrant 3) and black (quadrant 4) dots represent siRNAs that cause changes at or below this threshold cu [file pgen.1003112.s009.pdf]

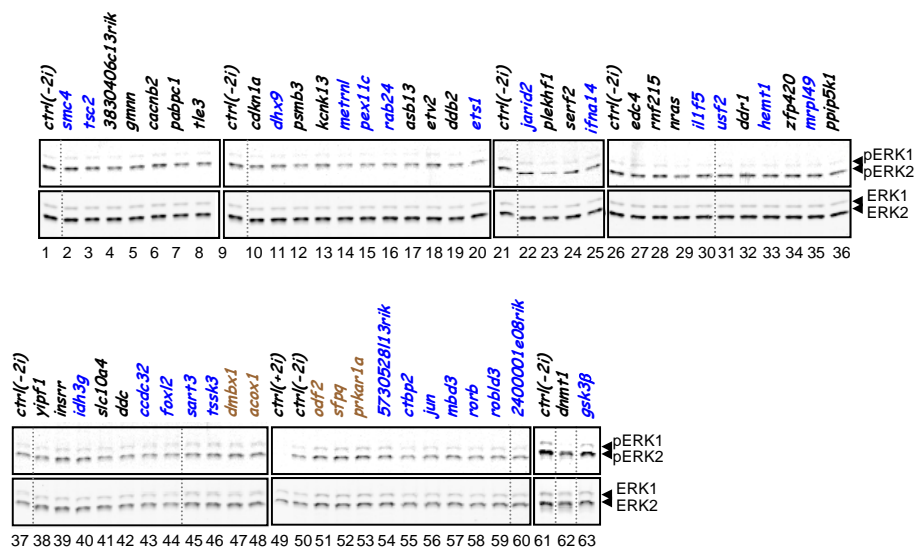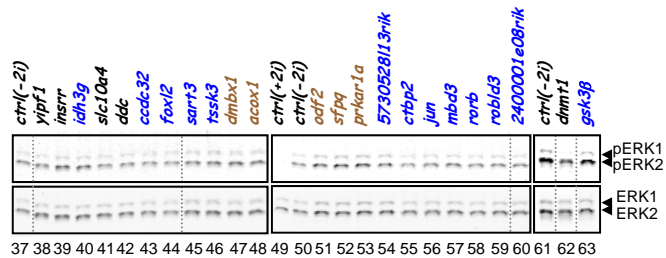

Supp. Fig. S10

Supplement: Figure S10 — Western blot analysis of phospho-ERK levels upon depletion of genes identified in the secondary siRNA screens. ERK activation levels following 2i withdrawal (−2i) for 20 mins were determined by western blotting using a phospho-ERK-specific antibody (pERK; top panels) and normalized against total ERK levels (bottom panels). siRNAs against the indicated genes from the “1i” screens (ERK hits in black, GSK3 hits in brown and ERK/GSK3 hits in blue) or control non-targeting siRNAs (ctrl) are indicated. The presence or absence of inhibitors in the control samples is indicated (+/−2i). Dotted lines indicate where gels have been cut to remove irrelevant lanes and rejoined. The data shown are from one of three biological replicate experiments. (PDF) [file pgen.1003112.s010.pdf]

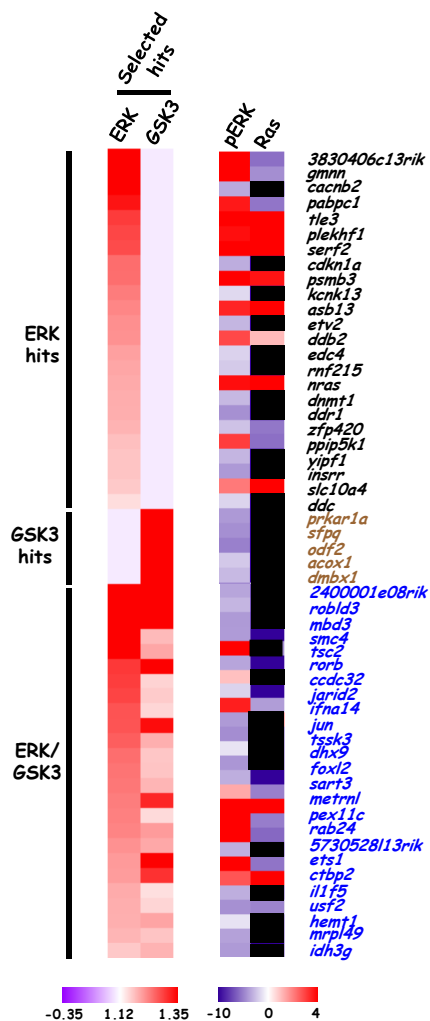

Supp. Fig. S11

Supplement: Figure S11 — Summary of the effects of each screen hit on the activity of Ras and ERK. A heatmap summary (see Figure 2B for details) is shown on the left illustrating the magnitude of the effects of the selected screen hits in each of the “1i” screens. These selected hits are distributed within three categories as indicated on the heatmap. Genes are ranked according to their effect in the “1i” screen where only the MEK inhibitor was removed. The heatmap on the right illustrates the magnitude of effect of depletion of each of the indicated genes on the levels of phospho-ERK (pERK) or the activity of Ras (see Figure 4). The colour code shown below relates to the magnitude of change above or below the mean of the control siRNAs (calculated as number of standard deviations from the mean). Black lines indicate that the effect was not assayed in the Ras activation assay. (PDF) [file pgen.1003112.s011.pdf]

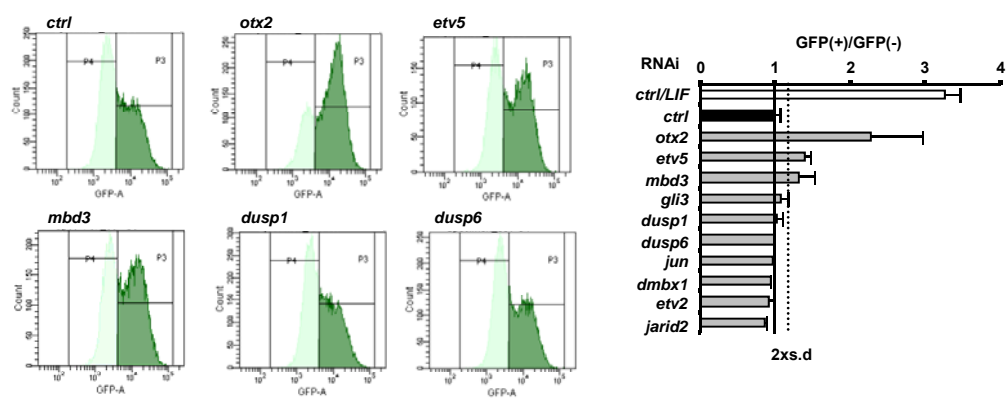

Supp. Fig. S12

Supplement: Figure S12 — GFP expression level changes upon gene depletion under LIF conditions. FACS profiles of GFP expression in Rex1GFPd2 ES cells grown in the presence of LIF and released for 28 hrs. The profiles corresponding to high GFP expressing cells are shown in dark green. The effect of pre-treating cells with control non-targeting siRNAs or siRNAs against the indicated genes on the GFP expression profile under these conditions is shown. The ratio of high to low GFP expressing cells is shown quantitatively on the right as means ± SEM; n = 2–3). The vertical lines represent the mean of the non-targeting control (ctrl) (solid line) and two standard deviations above this mean (dotted line). (PDF) [file pgen.1003112.s012.pdf]

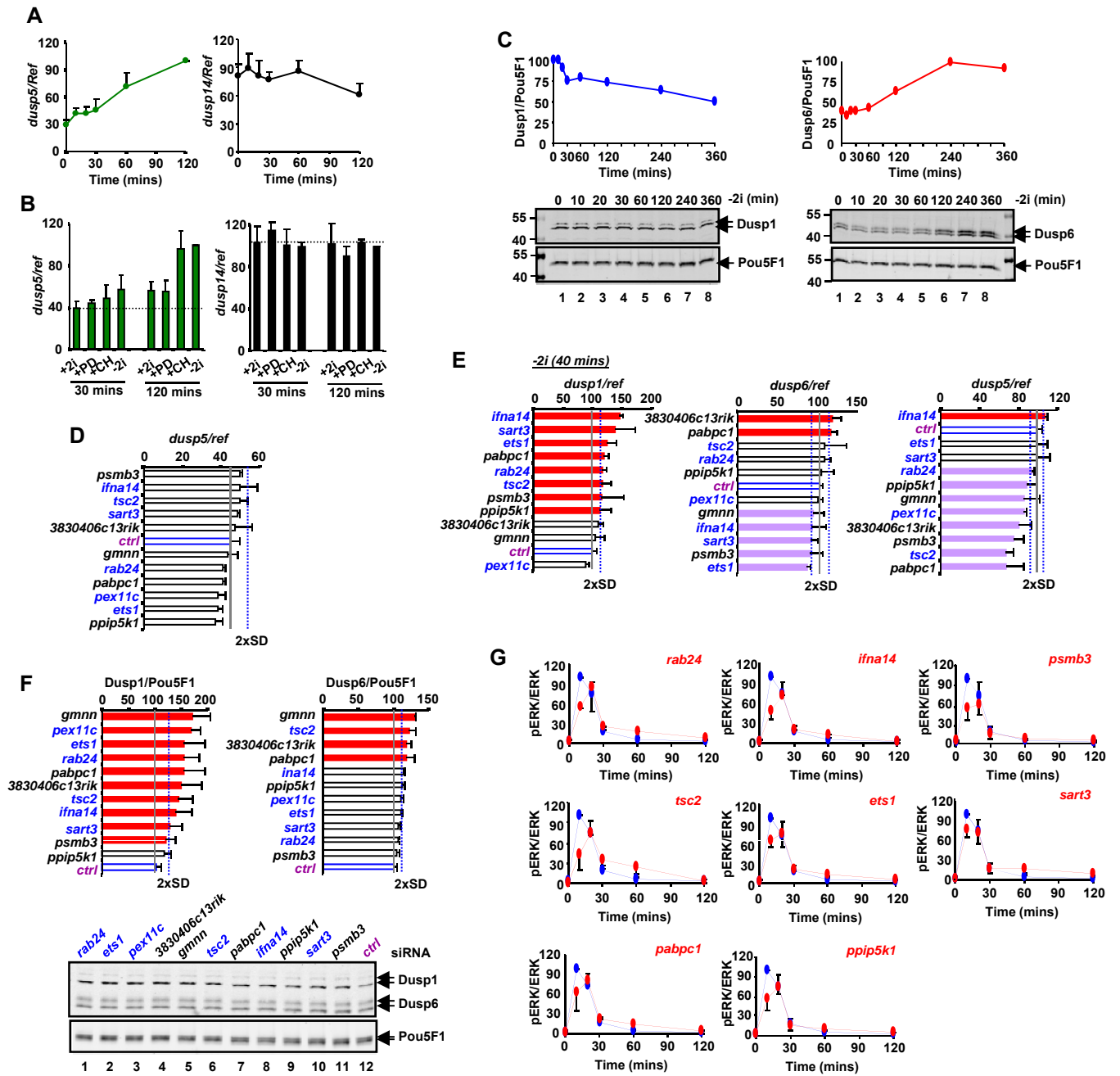

Supp. Fig. S13

Supplement: Figure S13 — The regulation of Dusp activity and ES cell differentiation (A–B and D–E) RT-qPCR analysis of dusp1, dusp5, dusp6 and dusp14 mRNA expression in mouse ES cells. Data are normalised by the average values of three reference genes (ref) and are presented as means ± SEM and are the average of three biological replicates (n = 3). (A) The kinetics of dusp5 and dusp14 expression at the indicated times following 2i withdrawal. (B) The effects of the indicated inhibitors, either alone or in combination, on the expression of dusp5 and dusp14 at the indicated times following inhibitor withdrawal. (D and E) The effects of depletion of the indicated genes on dusp5 mRNA expression in the presence, (D) or dusp1, dusp5, or dusp5 mRNA expression in the absence, (E) of 2i. The blue dashed lines indicate the threshold level (2× SD above/below the mean of the negative controls) and levels below and above this are indicated by red and pink bars, respectively. The average activity in the presence of control siRNA (ctrl) is shown by the solid grey line. (C and F) Dusp1 and Dusp6 protein expression measured by western blot analysis (bottom panels) was quantified and normalized by Pou5F1 levels (shown graphically in the top panels). (C) The kinetics of Dusp1 and Dusp6 expression at the indicated times following 2i withdrawal. (F) The effects of depletion of the indicated genes on Dusp1 and Dusp6 expression in the presence of 2i. Data are the average of two experiments. (G) Active ERK levels were determined by the ratio of phospho-ERK (pERK)/total ERK (ERK) levels at the indicated times following 2i release for the indicated times in the presence of the indicated siRNAs (red lines) or control siRNA (blue lines). The data are plotted relative to maximal levels with the control siRNA (taken as 100) and are presented as means ± SEM from the average of two biological replicates (n = 2). (PDF) [file pgen.1003112.s013.pdf]

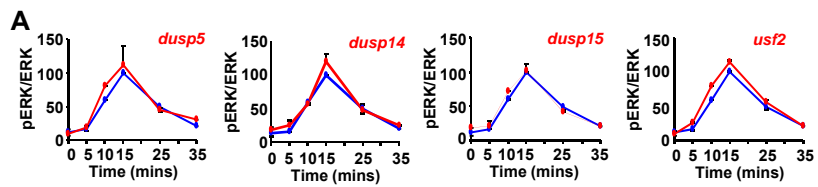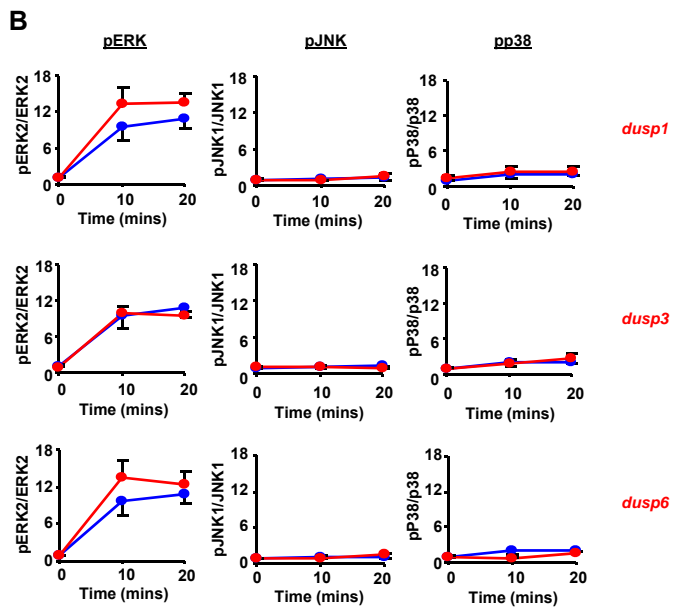

Supp. Fig. S14

Supplement: Figure S14 — The role of Dusps in MAP kinase activation in ES cells. (A) Active ERK levels were determined by the ratio of phospho-ERK (pERK)/total ERK (ERK) levels at the indicated times following 2i release in the presence of the indicated siRNAs (red lines) or control siRNA (blue lines). The data are plotted relative to maximal levels with the control siRNA (taken as 100) and are presented as means ± SEM from the average of two biological replicates (n = 2). (B) Active levels of the indicated MAPKs were determined by the ratio of phosphorylated (p) to non-phosphorylated forms at the indicated times following 2i release in the presence of the indicated siRNAs (red lines) or control siRNA (blue lines). The data are plotted relative to basal levels with the control siRNA in the presence of 2i (taken as 1) and are presented as means ± SEM from the average of two biological replicates (n = 2). (PDF) [file pgen.1003112.s014.pdf]
