# Supplementary material for: A Genome-Wide RNAi Screen Reveals MAP Kinase Phosphatases as Key ERK Pathway Regulators during Embryonic Stem Cell Differentiation
Source: PLoS Genet. 2012 Dec 13;8(12):e1003112. doi: 10.1371/journal.pgen.1003112 (PMC3521700; doi:10.1371/journal.pgen.1003112)
Supplement: Table S3 — Overlaps with other siRNA screens. Overlaps between genes identified in this paper as important for promoting loss of pluripotency and onset of differentiation (Table S4) or the maintenance of pluripotency (Table S2) and previous genome-wide and focused siRNA screens conducted in mouse ES cells. (PDF) [file pgen.1003112.s017.pdf]

**Differentiation**	***Fazzio et al (2008)***	
	Offical Gene symbol	***RefSe_mRNA***
	Mbd3	NM_013595

**Differentiation**	***Ding et al (2009)***	
	Offical Gene symbol	***RefSe_mRNA***
	Psmd14	NM_021526
	Zfp420	NM_172740

**Differentiation**	***Abujarour et al (2010)***	
	Offical Gene symbol	***RefSe_mRNA***
	Jun	NM_010591
	Nt5c	NM_015807

**Differentiation**	***Kagey et al (2010)***	
	Offical Gene symbol	***RefSe_mRNA***
	Bcor	NM_029510
	Cnot8	NM_026949
	Ctbp2	NM_009980
	Etv5	NM_023794
	Fhl4	NM_010214
	Foxk1	NM_199068
	Foxo1	NM_019739
	Jun	NM_010591
	Mbd3	NM_013595
	Med20	NM_020048
	Pou6f2	NM_175006

**Differentiation**	***Westerman et al (2011)***	
	Offical Gene symbol	***RefSe_mRNA***
	Robld3 (MP1)	NM_031248

**Pluripotency**	***Fazzio et al (2008)***	
	Offical Gene symbol	***RefSe_mRNA***
	Stat3	NM_011486
	Smc1a	NM_019710

**Pluripotency**	***Ding et al (2009)***	
	Offical Gene symbol	***RefSe_mRNA***
	Ctr9	NM_009431
	Dusp3	NM_028207
	Stat3	NM_011486
	Wdr5	NM_080848
	Zbtb20	NM_019778

**Pluripotency**	***Hu et al (2009)***	
	Offical Gene symbol	***RefSe_mRNA***
	Ctr9	NM_009431
	Eif4a1	NM_144958
	**Smc1a**	NM_019710

**Pluripotency**	***Abujarour et al (2010)***	
	Offical Gene symbol	***RefSe_mRNA***
	Wdr5	NM_080848

**Pluripotency**	***Kagey et al (2010)***	
	Offical Gene symbol	***RefSe_mRNA***
	Esrrb	NM_011934
	Myst2	NM_177619
	Smc1a	NM_019710
	Smc3	NM_007790

Supplementary Table S3. Overlaps with other siRNA screens
