# Supplementary material for: A Genome-Wide RNAi Screen Reveals MAP Kinase Phosphatases as Key ERK Pathway Regulators during Embryonic Stem Cell Differentiation
Source: PLoS Genet. 2012 Dec 13;8(12):e1003112. doi: 10.1371/journal.pgen.1003112 (PMC3521700; doi:10.1371/journal.pgen.1003112)
Supplement: Table S6 — Summary of the point of action of siRNAs with respect to the ERK signaling pathway. Genes targeted by the siRNA constructs are grouped according to whether they act to potentiate from Ras activity, act downstream of Ras to activate ERK or play a role downstream from, or in parallel to, ERK signalling. Each column indicates the association of the genes with the ERK, GSK or both pathways, as determined by the “1i” screens (see Figure S5). (PDF) [file pgen.1003112.s020.pdf]

**Upstream from RAS activation**		
**ERK only hits**	**ERK/GSK hits**	**GSK3 only hits**
slc10a4	*ccdc32*	
tle3	*metrnl*	
asb13	*ctbp2*	
plekhf1		
nras		
serf2		
psmb3		
ddb2		
**Upstream from ERK activation**		
**ERK only hits**	**ERK/GSK hits**	**GSK3 only hits**
3830406c13rik	*rab24*	
gmnn	*ets1*	
pabpc1	*pex11c*	
ppip5k1	*tsc2*	
	ifna14	
	sart3	
**Downstream from ERK activation**		
**ERK only hits**	**ERK/GSK hits**	**GSK3 only hits**
kcnk13	*il1f5*	*prkar1a*
cacnb2	*tssk3*	*sfpq*
ddc	*robld3*	*odf2*
insrr	*gsk3β*	*acox1*
ddr1	*mrpl49*	*dmbx1*
cdkn1a	*idh3g*	
yipf1	*hemt1*	
zfp420	*5730528l13rik*	
rnf215	*2400001e08rik*	
etv2	*pou5f1*	
dnmt1	*jun*	
edc4	*rorb*	
	foxl2	
	usf2	
	mbd3	
	jarid2	
	smc4	
	dhx9	

Table S4. Summary of the point of action of siRNAs with respect to the ERK signaling pathway.
