# Supplementary material for: A Genome-Wide RNAi Screen Reveals MAP Kinase Phosphatases as Key ERK Pathway Regulators during Embryonic Stem Cell Differentiation
Source: PLoS Genet. 2012 Dec 13;8(12):e1003112. doi: 10.1371/journal.pgen.1003112 (PMC3521700; doi:10.1371/journal.pgen.1003112)
Supplement: Table S7 — PCR primers used in RT-qPCR. The sequences of both forward (F) and reverse (R) primers are provided. (PDF) [file pgen.1003112.s021.pdf]

Oligo number	Oligo name	RefseqId	gene symbol
ES14_F	fgf5_F	NM_010203	*fgf5*
ES14_R	fgf5_R	NM_010203	*fgf5*
ES41_F	rex1_F	NM_009556	*rex1, zfp42*
ES41_R	rex1_R	NM_009556	*rex1, zfp42*
ES45_F	dusp1_F	NM_013642	*dusp1*
ES45_R	dusp1_R	NM_013642	*dusp1*
ES51_F	dusp5_F	NM_001085390	*dusp5*
ES51_R	dusp5_R	NM_001085390	*dusp5*
ES48_F	dusp6_F	NM_026268	*dusp6*
ES48_R	dusp6_R	NM_026268	*dusp6*
ES49_F	dusp14_F	NM_019819	*dusp14*
ES49_R	dusp14_R	NM_019819	*dusp14*
ES38_F	gapdh_F	NM_008084	*gapdh*
ES38_R	gapdh_R	NM_008084	*gapdh*
ES39_F	tbp_F	NM_013684	*tbp*
ES39_R	tbp_R	NM_013684	*tbp*
ES40_F	hmbs_F	NM_001110251	*hmbs*
ES40_R	hmbs_R	NM_001110251	*hmbs*
ES15_F	nestin_F	NM_016701	*nestin*
ES15_R	nestin_R	NM_016701	*nestin*
ES18_F	gata4_F	NM_008092	*gata4*
ES18_R	gata4_R	NM_008092	*gata4*
ES23_F	tbx6_F	NM_011538	*tbx6*
ES23_R	tbx6_R	NM_011538	*tbx6*

Supplementary Table S5. PCR primers

Sequence	location(exons)	product size
5' GATGGCAAAGTCAATGGCTC 3'	1	
5' GTAAATTTGGCACTTGCATGG 3'	2&3	154
5' GAAATGGACTAAGAGCTGGGA 3'	1	
5' TGAACAATGCCTATGACTCAC 3'	4	212
5' CCAAGGAGGATATGAAGCGT 3'	1&2	
5' CAGGTACAGGAAGGACAGGA 3'	3	186
5' TGAAATCCTTCCCTTCCTCTACC 3'	3	
5' TTGCCTCCTTCTTCCCTGAC 3'	4	233
5' CAGCGACTGGAATGAGAACAC 3'	1	
5' GGAACTTACTGAAGCCACCT 3'	1&2	112
5' CCTGGAAATCCTTAGCACGA 3'	2&3	
5' CAATGCCTCCTATGTCTCCC 3'	3	196
5' GCACAGTCAAGGCCGAGAA 3'	3	
5' GCCTTCTCCATGGTGGTGA 3'	3	151
5' TCTTGGCTGTAAACTTGACCT 3'	4	
5' CTGGATTGTTCTTCACTCTTGG 3'	5&6	173
5' TCCTGAAACTCTGCTTCGCT 3'	11	
5' ACAGTTGCCCATCTTTCATCAC 3'	12&13	109
5' CTACATACAGGACTCTGCTGG 3'	2&3	
5' GTCTTCAGAAAGGCTGTCAC 3'	4	222
5' GAACCTGAATAAATCTAAGACGCC 3'	5	
5' TGTGCCCATAGTGAGATGAC 3'	6	166
5' TCCTTCCGATTTCCTGAGAC 3'	4	
5' CCGAAGTTTCCTCTTCACAC 3'	6	161
